# Dectin-1-activated dendritic cells trigger potent antitumour immunity through the induction of Th9 cells

**DOI:** 10.1038/ncomms12368

**Published:** 2016-08-05

**Authors:** Yinghua Zhao, Xiao Chu, Jintong Chen, Ying Wang, Sujun Gao, Yuxue Jiang, Xiaoqing Zhu, Guangyun Tan, Wenjie Zhao, Huanfa Yi, Honglin Xu, Xingzhe Ma, Yong Lu, Qing Yi, Siqing Wang

**Affiliations:** 1 Department of Cancer Immunology, Institute of Translational Medicine, The First Hospital of Jilin University, Changchun 130061, China; 2 Cancer Center of the First Hospital of Jilin University, Changchun 130061, China; 3 The First Hospital and Institute of Immunology, Jilin University, Changchun 130061, China; 4 Laboratory of Virology, National Vaccine and Serum Institute, Beijing 100176, China; 5 Department of Cancer Biology, Lerner Research Institute, Cleveland Clinic, Cleveland, Ohio 44195, USA

## Abstract

Dectin-1 signalling in dendritic cells (DCs) has an important role in triggering protective antifungal Th17 responses. However, whether dectin-1 directs DCs to prime antitumour Th9 cells remains unclear. Here, we show that DCs activated by dectin-1 agonists potently promote naive CD4^+^ T cells to differentiate into Th9 cells. Abrogation of dectin-1 in DCs completely abolishes their Th9-polarizing capability in response to dectin-1 agonist curdlan. Notably, dectin-1 stimulation of DCs upregulates TNFSF15 and OX40L, which are essential for dectin-1-activated DC-induced Th9 cell priming. Mechanistically, dectin-1 activates Syk, Raf1 and NF-κB signalling pathways, resulting in increased p50 and RelB nuclear translocation and TNFSF15 and OX40L expression. Furthermore, immunization of tumour-bearing mice with dectin-1-activated DCs induces potent antitumour response that depends on Th9 cells and IL-9 induced by dectin-1-activated DCs *in vivo*. Our results identify dectin-1-activated DCs as a powerful inducer of Th9 cells and antitumour immunity and may have important clinical implications.

## Abstract (web summary)

Dendritic cells instruct different types of T cell responses depending on the types of microbial ligands sensed. Here the authors show that dendritic cells stimulated via Dectin-1 receptor upregulate TNFSF15 and OX40L and induce T helper 9 response and efficient antitumour immunity in mouse models.

## Graphical abstract

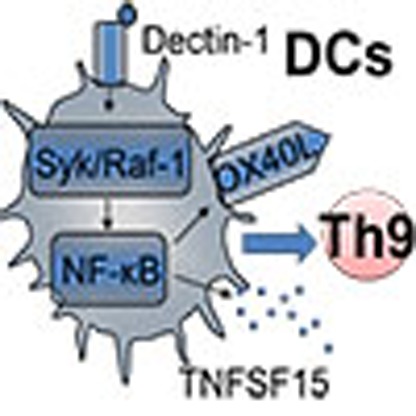

Naive CD4^+^ T cells, on antigenic activation, differentiate into various T helper (Th) cell subsets, such as Th1, Th2, Th17 and T regulatory cells (Tregs)^1^,^2^. Th9 is a recently described Th cell subset characterized by the secretion of interleukin (IL)-9 (refs 3, 4). Th9 cells and IL-9 (Th9/IL-9) are pro-inflammatory and appear to function in a broad spectrum of autoimmune diseases and allergic inflammation^5^,^6^. We and others have recently reported that adoptive transfer of Th9 cells induces potent therapeutic immunity against melanoma tumours in mice, better than other Th cells^7^,^8^. We have shown that Th9-derived IL-9 is critical in promoting an efficient host CD8^+^ CTL-mediated antitumour immune response^8^,^9^. Th9-derived IL-9 was also shown to activate mast cells, which may contribute to Th9 cell-induced antitumour activities^7^. IL-9 has the potential to enhance the survival and proliferation of antitumour effector T cells^10^. These seminal findings provide an impetus for further investigation of efficient strategies to induce and expand Th9 cells for tumour immunotherapy.

Th9 cells can be generated *in vitro* by TGF-β and IL-4 in the presence of anti-CD3/CD28 antibodies^3^,^4^. However, mechanisms of Th9 cell differentiation under physiological and pathological conditions are poorly understood. Previous investigations showed that IL-1, IL-2, OX40L, TSLP and IL-25 promoted Th9 cell development^11^,^12^,^13^,^14^,^15^,^16^. However, these factors are not specific for Th9 differentiation because they are also associated with the development of Th1, Th2 and Th17 cells^17^,^18^,^19^,^20^,^21^. These investigations suggest that the initiation of Th9 cells depends on some specific profiles of cytokine and costimulatory signals.

Dendritic cells (DCs) are professional antigen-presenting cells (APCs) and play a crucial role in the induction of Th cells^22^,^23^. Dectin-1, a C-type lectin receptor, is expressed mainly by DCs, macrophages and neutrophils^24^,^25^. DCs sense fungal pathogens through dectin-1, which recognizes β-1-3-glucans present on the fungal cell wall, and trigger the host immune response against fungal pathogens^26^. Dectin-1 triggers Syk and Raf1 downstream signalling pathways, which subsequently regulate the activation of canonical and noncanonical NF-κB pathways^24^. Dectin-1 activation in DCs stimulates the secretion of IL-6, TNF-α and IL-12p40, which polarize naive CD4^+^ T cells into Th17 and Th1 cells, the key effector cells for antifungal immunity^27^,^28^. However, whether dectin-1 activation in DCs favours the induction of antitumour Th9 cells remains unclear.

In this study, we found that dectin-1 activation in DCs potently promotes the induction of Th9 cells. We show that dectin-1 signalling stimulates DCs to overexpress TNFSF15 and OX40L, which are responsible for promoting Th9 cell differentiation primed by dectin-1-activated-DCs *in vitro*. Syk, Raf1 and NF-κB signalling pathways triggered by dectin-1 are required for dectin-1-induced expression of TNFSF15 and OX40L. Furthermore, immunization of mice bearing melanoma or myeloma tumours with dectin-1-activated DCs induces potent antitumour responses that depend on Th9 cells and IL-9. Our results thus identify dectin-1-activated DCs as a powerful inducer of Th9 cells and antitumour immunity and may have important clinical implications.

## Results

### Dectin-1-activated DCs enhance Th9 cell priming *in vitro*

**Figure 1:**
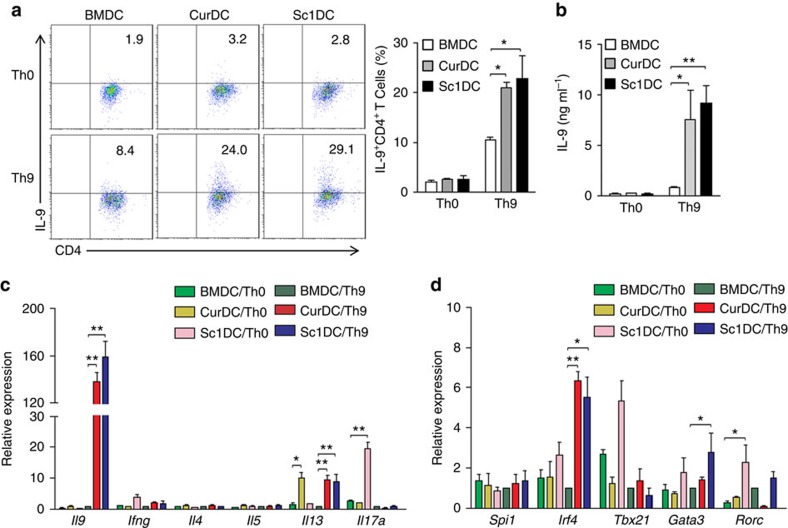
Dectin-1-activated DCs enhance Th9 cell differentiation *in vitro*. Naive CD4^+^ T cells from spleens of mice (*n*=3–5) were cocultured with DCs matured with TNF-α/IL-1β (BMDC), Curlan (CurDC) or Scleroglucan (SclDC) in the presence of anti-CD3 with (Th9) or without (Th0) addition of Th9-polarizing cytokines TGF-β and IL-4 for 3 days. Culture supernatants and CD4^+^ T cells separated by the magnetic cell sorting (MACS) were collected for analysis. (**a**) Cells were stained with anti-CD4 and anti-IL-9 antibodies and subjected to flow cytometry analysis. Numbers in the dot plots represent the percentages of CD4^+^IL-9^+^ T cells. Right, summarized results of four independent experiments obtained as at left. (**b**) ELISA assessed the IL-9 secretion in the cocultures. (**c**,**d**) qPCR analysis of Th9-, Th1-, Th2- and Th17-related cytokines (**c**) and transcription factors (**d**). Expression was normalized to *Gapdh* and set at 1 in BMDC-induced Th9 cells. Results shown are the mean±s.d. of 3–5 independent experiments. *P<0.05; **P<0.01 (Student's *t*-test).

Mouse immature DCs (iDCs) expressed dectin-1 (Supplementary Fig. 1). To address whether dectin-1-activated DCs affected the differentiation of Th9 cells, we matured mouse bone marrow (BM)-derived DCs with TNF-α plus IL-1β (BMDCs) or a selective dectin-1 agonist Curdlan (CurDCs) and stimulated naive CD4^+^ T cells under Th9-polarizing conditions (without anti-CD28 antibody) with BMDCs or CurDCs. We found that CurDCs efficiently enhanced the development of IL-9-producing Th9 cells (Fig. 1a) and increased IL-9 production at both mRNA and protein levels as compared with BMDCs (Fig. 1b,c). Furthermore, Th9 cells primed by CurDCs expressed significantly higher levels of Th9-related transcription factor *Irf4* than those primed by BMDCs (Fig. 1d). We also examined the expression of Th1-, Th2- and Th17-related cytokines and transcription factors and found that Th9 cells primed by CurDCs did not express most of the Th1-, Th2- and Th17-related cytokines and transcription factors, such as *Ifng*, *Il4*, *Il5*, *Il17*, *Tbx21* and *Rorc* (Fig. 1c,d), although the Th2-related cytokine *Il-13* was slightly increased (Fig. 1c). This result demonstrated that CurDCs reinforced Th9 cell differentiation.

To confirm the capability of dectin-1-activated DCs in polarizing naive CD4^+^ T cells into Th9 cells, we used another dectin-1 agonist Scleroglucan to mature mouse DCs (SclDCs). Similarly, as compared with BMDCs, SclDCs significantly enhanced Th9 cell differentiation (Fig. 1a), leading to higher expression of IL-9, *Irf4*, *Il13* and the Th2-related transcription factor *Gata3* (Fig. 1b–d), whereas the expression of other Th-related cytokines and transcription factors remained unchanged (Fig. 1c,d).

To examine the role of dectin-1 signalling in activating naturally occurring DCs in Th9 differentiation, mouse spleen CD11c^+^ cells were isolated, activated by curdlan and cocultured with T cells. Similarly, Curdlan-treated natural DCs drove Th9 differentiation by enhancing Th cell *Il9* expression as compared with untreated natural DCs (Supplementary Fig. 2).

Next we analysed the effects of dectin-1-activated DCs on other Th cell differentiation. Naive CD4^+^ T cells were cocultured with BMDCs, CurDCs or dectin-1^−/−^CurDCs under Th1-, Th2-, Th17- and Treg-polarizing conditions. As compared with BMDCs, CurDCs moderately enhanced Th1 and Th17 differentiation by increasing *Ifng*, *Tbx21*, *Il17a* and *Rorc* expression, respectively (Supplementary Fig. 3); while dectin-1^−/−^ CurDC-induced Th1 and Th17 cells expressed less *Ifng* and *Il17a* than CurDC-induced Th cells, respectively (Supplementary Fig. 3). Together, these results demonstrated the potency of dectin-1-activated DCs in the induction of Th9 cells.

### Th9 induction by curdlan-activated DCs relies on dectin-1

**Figure 2:**
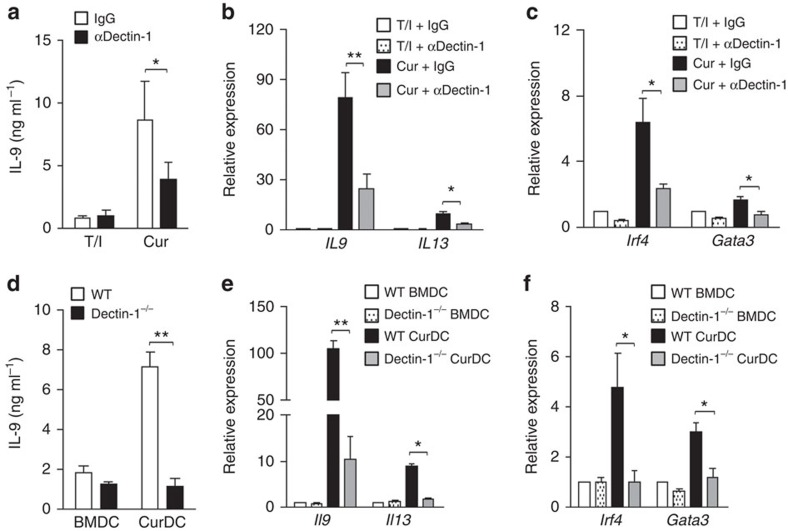
Abrogation of dectin-1 inhibits the capability of DCs to prime Th9 cells *in vitro*. iDCs generated from mice (*n*=3–5) were matured with TNF-α/IL-1β (T/I) or Curdlan (Cur) in the presence of either a blocking anti-dectin-1 antibody (αDectin-1) or an isotype control IgG (IgG) for 2 days and cocultured with CD4^+^ naive T cells under Th9 polarizing conditions for 3 days. (**a**) ELISA assessed IL-9 secretion in the cocultures. qPCR assessed the mRNA levels of the indicated Th cytokines (**b**) and transcription factors (**c**) in CD4^+^ T cells from the cocultures. BMDC and CurDC generated from WT- or dectin-1^−/−^ mice (*n*=3) were subjected to culture with naive CD4^+^ T cells under Th9 polarizing conditions. (**d**) ELISA assessed IL-9 secretion in the cocultures. (**e**,**f**) Same as in **b** and **c**, the expression of the indicated genes in CD4^+^ T cells was examined by qPCR. Results shown are the mean±s.d. of 3–5 independent experiments. *P<0.05; **P<0.01 (Student's *t*-test).

To explore the contribution of dectin-1 to dectin-1-activated DC-induced Th9 cell differentiation, mouse DCs matured with Curdlan plus a dectin-1 blocking antibody (αDectin-1) were used to prime Th9 cells. While Th9 cells primed by αDectin-1-treated BMDCs expressed comparable levels of IL-9, *Irf4*, *Il13* and *Gata3* as compared with those primed by BMDCs (Fig. 2a–c), Th9 cells primed by αDectin-1-treated CurDCs expressed significantly lower levels of IL-9, *Irf4*, *Il13* and *Gata3* than those primed by CurDCs (Fig. 2a–c). This result indicated that dectin-1 played an important role in directing DCs for Th9 cell induction.

To further confirm the function of dectin-1 in activating DCs for Th9 cell induction, we generated BMDCs and CurDCs from wildtype (WT) and dectin-1 knockout (dectin-1^−/−^) mice and used them to prime Th9 cells. Dectin-1^−/−^ CurDCs failed to enhance Th9 cell differentiation, as the expression of IL-9 and *Irf4* was almost completely abolished in Th9 cells primed by dectin-1^−/−^ CurDCs compared with WT CurDCs (Fig. 2d–f). Dectin-1-deficiency did not affect BMDCs in priming Th9 cells, as demonstrated by the similar expression levels of IL-9 mRNA and IL-9 protein by Th9 cells primed by dectin-1^−/−^ BMDCs and WT BMDCs (Fig. 2d,e). Notably, Th9 cells primed by dectin-1^−/−^ CurDCs also expressed much lower levels of *Il13* and *Gata3* than those primed by WT CurDCs (Fig. 2d–f and Supplementary Fig. 4). Collectively, these data demonstrated the important role of dectin-1 in directing DCs for Th9 cell differentiation.

### Dectin-1 stimulates DCs to express TNFSF15 and OX40L

Th cell differentiation relies on specific profiles of cytokines and costimulatory molecules^1^,^2^. To explore the molecular mechanisms by which dectin-1-activated DCs drove Th9 differentiation, we performed gene expression profiling analyses in BMDCs versus CurDCs. Among 26,423 mouse genes, we identified 42 upregulated genes of cytokines, chemokines and costimulatory surface molecules that might be involved in Th cell activation, polarization and chemotaxis (Table 1).

**Figure 3:**
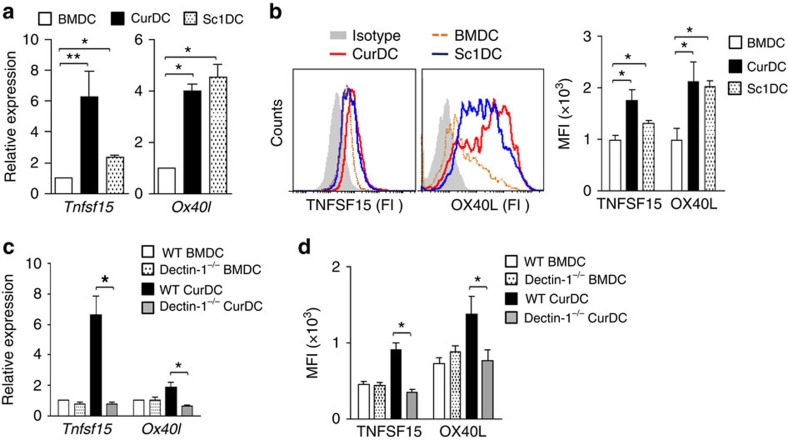
Dectin-1 activation in DCs induces TNFSF15 and OX40L expression. iDCs generated from mice (*n*=3–5) were matured by TNF-α/IL-1β (BMDC), Curdlan (CurDC) or Scleroglucan (SclDC) for 48 h. (**a**) qPCR analysed the mRNA levels of *Tnfsf15* and *Ox40l* in DCs. (**b**) Flow cytometry analysis of TNFSF15 and OX40L protein surface expression in DCs. Right, summarized results of three independent experiments obtained as the left. MFI, mean fluorescence intensity. BMDCs and CurDCs were generated from WT or dectin-1^−/−^ mice (*n*=3). Same as in **a** and **b**, the mRNA (**c**) and protein (**d**) levels of TNFSF15 and OX40L in DCs were analysed by qPCR and flow cytometry. Results shown are the mean±s.d. of three independent experiments. *P<0.05; **P<0.01 (Student's *t*-test).

Besides the cytokines *Il12p35* (*Il12a*), *Il12p40* (*Il12b*) and *Tnf* which were reportedly upregulated by dectin-1 activation^24^,^27^, our data identified additional new cytokines and costimulatory molecules that were upregulated by Curdlan stimulation, especially the TNF/receptor family members, including *Tnfsf15* (*Tl1a*), *Tnfsf4* (*Ox40l*), *Tnfsf8*, *Tnfrsf26* and *Tnfrsf12a* (Table 1). The upregulated expression of TNFSF15 and OX40L by dectin-1-activated DCs compared with BMDCs was confirmed by quantitative real-time PCR (qPCR) (Fig. 3a) and flow cytometry analysis (Fig. 3b). Furthermore, the upregulated expression of TNFSF15 and OX40L was completely abolished in CurDCs and SclDCs from dectin-1^−/−^ mice (Fig. 3c,d). Notably, Curdlan stimulation drove natural DCs to express TNFSF15 and OX40L *in vivo* (Supplementary Fig. 5). In addition, Curdlan stimulation led to upregulation of DC costimulatory surface proteins CD86, CD40 and CD80 as compared with the treatment with TNF-α/IL-1β (Supplementary Fig. 6), suggesting improved immunogenicity of dectin-1-activated DCs.

Together, these results demonstrated that dectin-1 activation stimulated DCs to express a specific profile of cytokines, chemokines and surface costimulatory molecules, especially the TNF/receptor family members, suggesting the potential mechanisms for dectin-1-activated DCs in the induction of Th9 cells.

### Dectin-1 directs Th9 priming via TNFSF15 and OX40L

**Figure 4:**
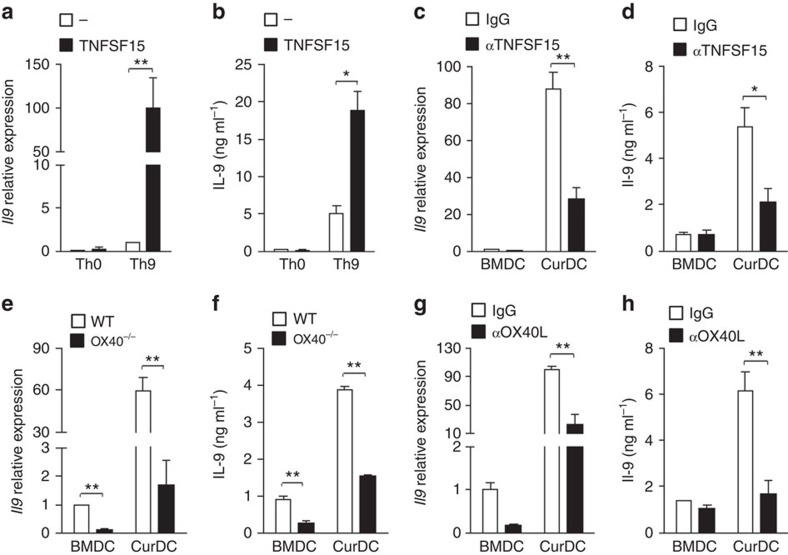
TNFSF15 and OX40L contribute to Th9 cell differentiation primed by dectin-1-activated DCs. (**a**,**b**) Pooled splenic CD4^+^ naive T cells from mice (*n*=3–5) were differentiated under Th0 or Th9 polarizing conditions with (TNFSF15) or without addition of TNFSF15 for 3 days. (**a**) qPCR assessed IL-9 expression in CD4^+^ T cells. (**b**) ELISA assessed IL-9 secretion in the culture. (**c**,**d**) Pooled splenic naive CD4^+^ T cells from mice (*n*=3) were cocultured with BMDCs or CurDCs under Th9 polarizing conditions with the addition of a TNFSF15-neutralization antibody (αTNFSF15) or isotype control IgG (IgG). Cells were cultured for 3 days. CD4^+^ T cells and culture supernatants were collected and subjected to qPCR (**c**) and ELISA (**d**) for IL-9 expression. (**e**,**f**) Pooled splenic naive CD4^+^ T cells from mice (*n*=3) were isolated from WT or OX40^−/−^ mice and differentiated under Th9 polarizing conditions in the presence of BMDCs or CurDCs for 3 days. CD4^+^ T cells and culture supernatants were collected for IL-9 expression by qPCR (**e**) and ELISA (**f**). Similar as **c** and **d**, pooled splenic naive CD4^+^ T cells from mice (*n*=3) were cocultured with BMDCs or CurDCs under Th9 polarizing conditions with the addition of an anti-OX40L blocking antibody (αOX40L) or control IgG (IgG). IL-9 expression was examined by qPCR (**g**) and ELISA (**h**). Results shown are the mean±s.d. of at least three independent experiments. *P<0.05; **P<0.01 (Student's *t*-test).

*Tnfsf15* was the top 4 among the 42 upregulated genes of cytokines, chemokines and surface costimulatory molecules in CurDCs (Table 1). We therefore investigated its role in mediating Th9 differentiation primed by dectin-1-activated DCs. First, we examined the function of TNFSF15 in Th9 cell differentiation. As shown in Fig. 4a,b, the addition of TNFSF15 significantly increased IL-9 mRNA and protein expressions by Th9 cells. To investigate the contribution of TNFSF15 to Th9 cell differentiation primed by dectin-1-activated DCs, a TNFSF15 neutralizing antibody (αTNFSF15) was used. The addition of αTNFSF15 significantly inhibited IL-9, *Irf4* and *Il13* expression in CurDC-induced Th9 cells as compared with the treatment of control IgG (Fig. 4c,d and Supplementary Fig. 7A,B); whereas the addition of αTNFSF15 did not affect IL-9 expression of BMDC-induced Th9 cells (Fig. 4c,d and Supplementary Fig. 7A,B). These results indicated that TNFSF15 contributed to Th9 cell differentiation primed by dectin-1-activated DCs.

Recent study has proposed a role of OX40/OX40L axis in Th9 cell differentiation^11^. Thus, we next sought to determine whether OX40L expressed by dectin-1-activated DCs is required for the Th9 cell differentiation primed by dectin-1-activated DCs. Naive CD4^+^ T cells were isolated from WT and OX40^−/−^ mice and differentiated into Th9 cells in the presence of BMDCs or CurDCs. CurDCs readily converted WT naive CD4^+^ T cells into Th9 cells (Fig. 4e,f and Supplementary Fig. 7C,D). However, the capability of CurDCs in converting OX40^−/−^ naive T cells into Th9 cells was largely abrogated as demonstrated by significantly lower IL-9 and *Irf4* expression by OX40^−/−^ Th9 cells primed by CurDCs (Fig. 4e,f and Supplementary Fig. 7C,D), indicating that OX40L contributed to Th9 cell development primed by dectin-1-activated DCs. Notably, CurDC-induced OX40^−/−^ Th9 cells also expressed less *Il13* than CurDC-induced WT Th9 cells (Supplementary Fig. 7C,D). To further explore the contribution of OX40L to dectin-1-activated DC-induced Th9 differentiation, an OX40L blocking antibody (αOX40L) was used. We found that the addition of αOX40L significantly diminished IL-9 expression by Th9 cells stimulated with CurDCs (Fig. 4g,h), demonstrating the important role of OX40L in dectin-1-activated DC-induced Th9 differentiation. To examine the effects of TNFSF15 and OX40L on the differentiation of other Th cells primed by dectin-1-activated DCs, αTNFSF15 and αOX40L were used during the induction of Th1, Th2, Th17 and Treg cells by CurDCs. Blocking TNFSF15 and OX40L slightly inhibited *Il17a* expression by CurDC-induced Th17 cells (Supplementary Fig. 8); whereas the addition of αTNFSF15 or αOX40L exhibited minor effects on the expression of *Ifng*, *Il4* and *Foxp3* by Th cells primed by CurDCs as compared with the addition of control IgG (Supplementary Fig. 8). Collectively, these results demonstrated that dectin-1-induced TNFSF15 and OX40L represented two Th9-polarizing signals from dectin-1-activated DCs.

### Dectin-1 induces TNFSF15 and OX40L via Syk and Raf1

**Figure 5:**
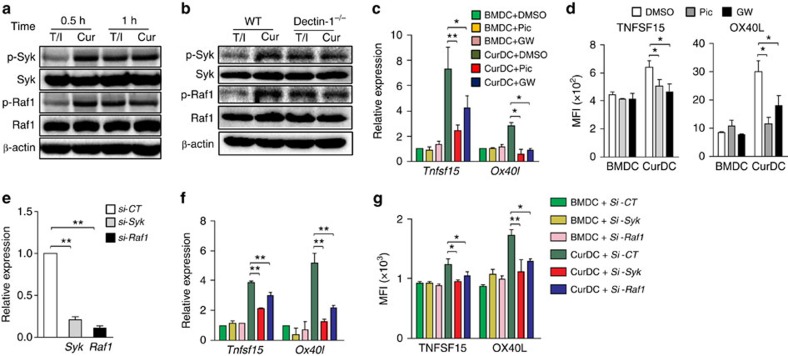
Dectin-1 induces TNFSF15 and OX40L expression through Syk and Raf1. (**a**) iDCs generated from mice (*n*=3–5) were stimulated with TNF-α/IL-1β (T/I) or Curdlan (Cur) for 0.5 or 1 h. Cell lysates were prepared and subjected to western blot analysis using indicated antibodies. (**b**) iDCs were prepared from WT and dectin-1^−/−^ mice (*n*=3) and stimulated with TNF-α/IL-1β or Curdlan for 0.5 h. Cell lysates were subjected to western blot analysis using indicated antibodies. iDCs were matured by TNF-α/IL-1β (BMDCs) or Curdlan (CurDCs) in the presence of piceatannol (Pic), GW5074 (GW) or DMSO as control for 48 h. (**c**) qPCR assessed *Tnfsf15* and *Ox40l* expression in DCs. (**d**) Flow cytometry of TNFSF15 and OX40L expression in DCs. Right, summarized results of three independent experiments obtained as at left. MFI, mean fluorescence intensity. (**e**) Mouse (*n*=3) iDCs were treated by *Syk* (*si-Syk*), *Raf1* (*si-Raf1*) or control siRNA (*Si-CT*) and subjected to the stimulation of TNF-α/IL-1β (BMDC) or Curdlan (CurDCs) for48 h. qPCR assessed mRNA levels of *Syk* and *Raf1* in DCs. (**f**,**g**) qPCR (**f**) and flow cytometry (**g**) analysed TNFSF15 and OX40L expression in DCs generated from mice (*n*=3). Data are representative of three (**a**,**b**) independent experiments or presented as mean±s.d. of at least three (**c**–**g**) independent experiments. *P<0.05; **P<0.01 (Student's *t*-test).

We next examined the downstream signalling pathways of dectin-1 that are responsible for TNFSF15 and OX40L expression in DCs. Given that dectin-1 is known to regulate the transcription factor NF-κB via the activation of Syk and/or Raf1-dependent signalling^24^, we examined whether these signalling pathways mediated dectin-1-induced TNFSF15 and OX40L expression. We first compared the effects of Curdlan versus TNF-α/IL-1β on the activation of Syk and Raf1 signalling pathways in mouse iDCs. Curdlan treatment induced rapid increases of phosphorylated (p)-Syk and p-Raf1 in DCs as compared with the treatment with TNF-α/IL-1β (Fig. 5a), indicating that Curdlan was more potent than TNF-α/IL-1β in activating Syk and Raf1 in DCs. To further confirm the involvement of dectin-1 in Syk and Raf1 activation, iDCs generated from WT or dectin-1^−/−^ mice were used. Dectin-1 deficiency remarkably reduced the expression of p-Syk and p-Raf1 in iDCs treated with Curdlan, whereas it did not affect the phosphorylation of Syk and Raf1 in iDCs treated with TNF-α/IL-1β (Fig. 5b), indicating that Curdlan induced dectin-1-dependent activation of Syk and Raf1 in DCs.

To investigate the role of Syk and Raf1 signalling in dectin-1-induced TNFSF15 and OX40L expression, Syk-specific inhibitor Piceatannol and Raf1-specific inhibitor GW5074 were used during DC maturation. The inhibition of Syk or Raf1 in DCs significantly decreased TNFSF15 and OX40L expression induced by Curdlan (Fig. 5c,d). In addition, by using small interfering RNAs (siRNAs) to specifically silence *Syk* or *Raf1* in mouse DCs (Fig. 5e), we found that knockdown of either *Syk* or *Raf1* in DCs reduced TNFSF15 and OX40L expression induced by Curdlan (Fig. 5f,g). To examine the effects of Syk and Raf1 signalling in directing dectin-1-activated DCs for Th cell differentiation, Piceatannol- and GW5074-treated CurDCs were used to prime Th cells. Piceatannol- or GW5074-treated CurDCs were less effective in the induction of Th9, Th1 and Th17 cells than CurDCs, as demonstrated by the decreased expression of *Il9*, *Ifrg* and *Il17a* by Th cells primed by Piceatannol- or GW5074-treated CurDCs compared with control CurDCs, (Supplementary Fig. 9). Together, these results demonstrated that dectin-1 induced TNFSF15 and OX40L expression through Syk and Raf1 signalling pathways.

### Dectin-1 induces OX40L and TNFSF15 expression via NF-κB

**Figure 6:**
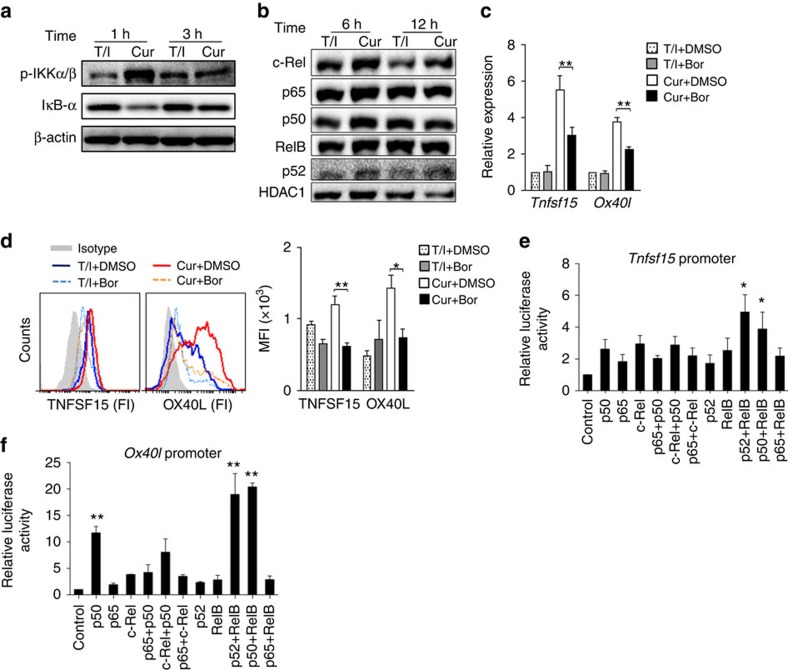
Dectin-1 induces TNFSF15 and OX40L expression through NF-κB signalling pathway. (**a**) Mouse (*n*=3–5) iDCs were stimulated with TNF-α/IL-1β (T/I) or Curdlan (Cur) for 1 and 3 h. Whole-cell lysates were prepared and subjected to western blot analysis using indicated antibodies. (**b**) Mouse (*n*=3) iDCs were stimulated with TNF-α/IL-1β (T/I) or Curdlan (Cur) for 6 or 12 h. Nuclear extracts were subjected to western blot analysis using indicated antibodies. HDAC1 was used as loading control. (**c**,**d**) Mouse (*n*=3) iDCs were matured by TNF-α/IL-1β (BMDC) or curdlan (CurDC) in the absence (DMSO) or presence of NF-κB inhibitor bortizomib (Bor) for 48 h. (**c**) qPCR analysed *Tnfsf15* and *Ox40l* expression in DCs. (**d**) Flow cytometry analysis of TNFSF15 and OX40L expression in DCs. Right, summary of results of three independent experiments obtained as at left. MFI, mean fluorescence intensity. (**e**,**f**) 293T cells were transfected with vectors contained *Tnfsf15* or *Ox40l* promoter or empty vector, followed by transfecting with vectors expressing the indicated NF-κB molecules. Luciferase reporter assay showed NF-κB-dependent activation of *Tnfsf15* (**e**) and *Ox40l* (**f**) promoter in 293T cells. Data are representative of at least three (**a**,**b**) independent experiments or presented as mean±s.d. of at least three (**c**–**f**) independent experiments. *P<0.05; **P<0.01 (Student's *t*-test).

To compare the effects of Curdlan versus TNF-α/IL-1β on the activation of NF-κB pathway, mouse iDCs were treated with TNF-α/IL-1β or Curdlan. Curdlan treatment increased the expression of p-IKKα/β and decreased that of IκB-α in mouse DCs (Fig. 6a), leading to a remarkable increase of c-Rel, p50 and RelB nuclear translocation and a slight increase of p65 and p52 nuclear translocation as compared with the treatment with TNF-α/IL-1β (Fig. 6b), indicating that Curdlan was more potent in activating NF-κB pathway in DCs than TNF-α/IL-1β. Bortezomib, a proteasome inhibitor, was used to inhibit NF-κB^14^. We found that mouse DCs matured by Curdlan plus bortezomib expressed less TNFSF15 and OX40L than those matured by Curdlan alone (Fig. 6c,d), indicating that dectin-1 induced TNFSF15 and OX40L expression through NF-κB signalling pathway.

To further determine the role of NF-κB pathway in dectin-1-induced TNFSF15 and OX40L expression, we performed luciferase reporter assays to examine whether these NF-κB molecules could bind directly to *Tnfsf15* and *Ox40l* promoters and affect their expression. We found that p50-RelB and p52-RelB dimmers could bind to and activate *Tnfsf15* promoter (Fig. 6e); while p50, p50-RelB and p52-RelB could bind to and activate *Ox40l* promoter (Fig. 6f). Considering that p52 nuclear translocation was only slightly increased by Curdlan treatment, we speculated that p50-RelB was responsible for dectin-1-induced TNFSF15 expression, while p50 and p50-RelB were responsible for dectin-1-induced OX40L expression. Collectively, these results demonstrated that dectin-1-induced TNFSF15 and OX40L expression was dependent on NF-κB signalling pathway.

### Dectin-1-activated DCs induce antitumour effects *in vivo*

**Figure 7:**
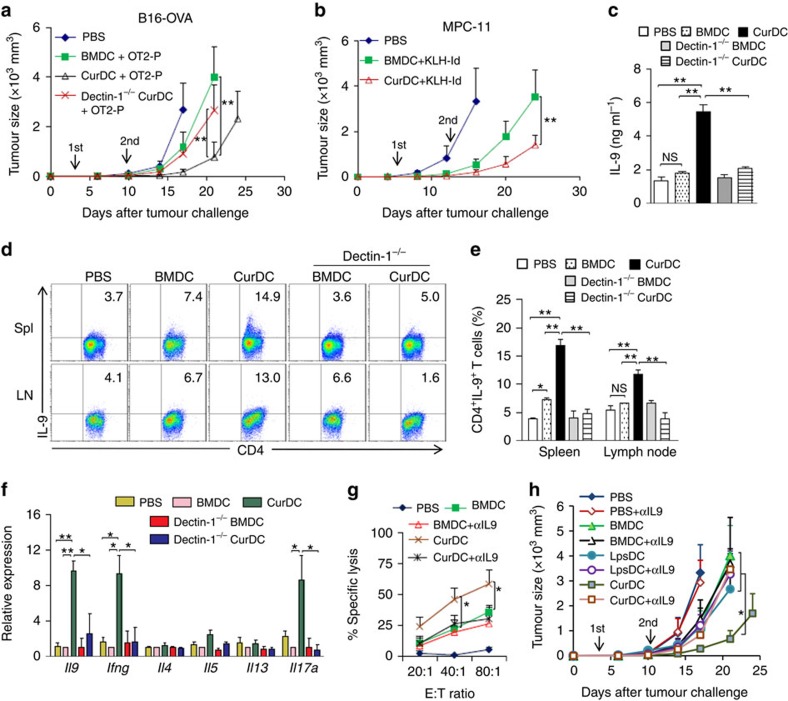
Role of Th9/IL-9 in dectin-1-activated DC-induced antitumour effects *in vivo*. (**a**) OT-II mice were injected subcutaneously with 1 × 10^5^ B16-OVA cells. On day 3 after tumour challenge, mice (*n*=10 per group) were given two weekly subcutaneously immunizations with 1 × 10^6^ OVA peptide-pulsed BMDCs or CurDCs generated from WT or dectin-1^−/−^ mice. Mice received PBS served as controls. Shown are the tumour growth curves. (**b**) Balb/c mice were injected subcutaneously with 1 × 10^6^ MPC-11 myeloma cells. On day 6 after tumour challenge, mice (*n*=10 per group) were given two weekly subcutaneously immunizations with 1 × 10^6^ KLH-Id-pulsed BMDCs or CurDCs. Mice received PBS served as controls. Shown are the tumour growth curves. (**c**–**f**) OT-II mice (*n*=4–5 per group) were injected subcutaneously with 1 × 10^5^ B16-OVA cells. On day 3 after tumour challenge, mice were immunized with OVA-peptide-pulsed BMDCs, CurDCs, dectin1^−/−^ BMDCs or dectin-1^−/−^ CurDCs. PBS served as control. On day 3 after the immunization, serum samples and total leukocytes from LNs and spleen cells were collected and restimulated with 5 μg ml^−1^ OVA_323-339_ peptides for 24 h. (**c**) ELISA analysis of serum IL-9. (**d**) Flow cytometry analysis of IL-9-producing CD4^+^ T cells. Numbers in the dot plots represent the percentages of CD4^+^IL-9^+^ T cells. (**e**) Summarized results of three independent experiments obtained in **d**. (**f**) qPCR analysis of the indicated genes. (**g**) Balb/c mice (*n*=3 per group) were immunized twice (1 week apart) with 1 × 10^6^ Id-KLH-pulsed BMDCs or CurDCs. PBS served as a control. Mice were given control IgG or an IL-9 neutralizing antibody (αIL9) every 3 days beginning 1 day after the first immunization. Results shown are MPC-11-specific cytotoxicity of spleen T cells from the mice. * shows *P*<0.05, comparing CurDC with BMDC, BMDC+αIL9 or CurDC+αIL9. (**h**) OT-II mice were injected subcutaneously with 1 × 10^5^ B16-OVA cells. On day 3 after tumour challenge, mice (*n*=10 per group) received two weekly immunizations with 1 × 10^6^ OVA peptide-pulsed BMDCs, LpsDCs or CurDCs. Mice received PBS served as controls. Mice were given control IgG or αIL9 every 3 days beginning 1 day after the first immunization. Shown are the tumour growth curves. * showing *P*<0.05, comparing CurDC with each of the five groups as indicated. In **a**–**g**, data are presented as mean±s.d. of two combined *in vivo* experiments (**a**,**b**) or at least three (**c**–**g**) independent *in vitro* experiments. In **h**, data from 10 mice per group are used. *P<0.05; **P<0.01 (Student's *t*-test).

To examine the role of dectin-1-activated DCs in tumour immunotherapy in mice, we generated BMDCs and CurDCs from WT and dectin-1^−/−^ mice, and pulsed them with OVA peptide (323–339). OT-II mice were immunized with OVA peptide-pulsed WT BMDCs, WT CurDCs or dectin-1^−/−^ CurDCs at days 3 and 10 after challenge with B16-OVA melanoma cells. Mice immunized with WT CurDCs displayed greater resistance to melanoma tumour growth than mice immunized with WT BMDCs, while WT BMDC vaccination showed moderate antitumour effects as compared with PBS control (Fig. 7a). Furthermore, idiotype (Id)-pulsed CurDCs also induced more potent anti-tumour response than Id-pulsed BMDCs in MPC-11 myeloma Balb/c mouse model (Fig. 7b). These results demonstrated that dectin-1-activated DCs induced potent therapeutic antitumour immunity *in vivo*. Notably, while blockade of TNFSF15 or OX40L by their specific antibodies partially inhibited the antitumour effects induced by CurDCs (Supplementary Fig. 10), dectin-1^−/−^ CurDC immunization was much less effective than WT CurDCs in inducing anti-melanoma response (Fig. 7a), indicating that CurDCs induced antitumour immunity in a dectin-1-dependent manner.

Notably, a very recent report by HyeMee *et al*. showed that the dectin-1 agonist Curdlan downregulated OX40L expression on myeloid DCs^29^, which is exactly the opposite of our finding that dectin-1 upregulated OX40L expression on bone marrow-derived myeloid DCs. The reasons for this discrepancy are unclear. We suggest that the different concentrations of Curdlan used for DC activation in these two studies (5 μg ml^−1^ in our study versus 10 μg ml^−1^ in HyeMee's study) may contribute to the difference of OX40L expression in myeloid DCs (Supplementary Fig. 11).

### Antitumour effects of dectin-1-activated DCs rely on Th9/IL-9

The observed important role of dectin-1-activated DCs in Th9 cell differentiation *in vitro* and the antitumour immunity *in vivo* prompted us to determine whether Th9 cells and IL-9 were involved in mediating the antitumour effects induced by dectin-1-activated DCs *in vivo*. To determine whether the immunization of dectin-1-activated DCs induces Th9 cells and IL-9 production, OT-II mice were immunized with OVA peptide-pulsed BMDCs or CurDCs. On day 3 after DC immunization, mice were killed, and mouse sera and total leukocytes from spleens and lymph nodes (LNs) were collected for the assessment of Th9 cell response. While no increase of serum IL-9 was observed in mice immunized with BMDCs as compared with mice treated with PBS (Fig. 7c), mice immunized with CurDCs showed significantly higher levels of serum IL-9 than mice receiving either BMDCs or PBS (Fig. 7c), indicating that dectin-1-activated DC immunization induced IL-9 production. Spleen and LN cells were restimulated with OT-II OVA peptides for 24 h before assay. Interestingly, intracellular staining detected significantly higher percentages of IL-9^+^CD4^+^ (Th9) cells in the spleen and LN cells from mice immunized with CurDCs than mice receiving BMDCs or PBS (Fig. 7d,e), though there was a modest increase of Th9 cells in the spleen and LN cells from mice immunized with BMDCs as compared with PBS control mice (Fig. 7d,e). qPCR and ELISA further confirmed the increase of IL-9 production in mice immunized with CurDCs compared with mice receiving BMDCs or PBS (Fig. 7f and Supplementary Fig. 12). These results demonstrated that dectin-1-activated DCs stimulated the production of Th9 cells and IL-9 *in vivo*. Dectin-1-activated DCs reportedly promoted the development of Th17 and Th1 cells^27^. In line with the published observations, we found that dectin-1-activated DC immunization upregulated the expression of *Ifng* and *Il17a* by spleen CD4^+^ cells as compared with the treatment with BMDCs or PBS (Fig. 7f and Supplementary Fig. 12).

To further confirm the role of dectin-1 signalling in activating DCs for the induction of Th9 cells and IL-9 production *in vivo*, we immunized OT-II mice with OVA peptide-pulsed WT CurDCs or dectin-1^−/−^ CurDCs. Mouse serum samples, spleen and LN cells were collected for the analysis of Th9 cell response. We found that dectin-1^−/−^ CurDC immunization failed to induce Th9 cell development in mice, since the serum IL-9, the number of IL-9^+^CD4^+^ (Th9) cells and the expression of *Il9* mRNA were completely abolished in mice immunized with dectin-1^−/−^ CurDCs as compared with those immunized with WT CurDCs (Fig. 7c–f).

Th9/IL-9 is known to induce tumour-specific CTL responses^8^,^30^. To address whether the induction of Th9/IL-9 by dectin-1-activated DCs *in vivo* resulted in potent antitumour CTL responses, Balb/c mice were immunized with Id-pulsed BMDCs or CurDCs. CurDC immunization induced higher levels of tumour-specific CTL activity than BMDCs (Fig. 7g). Notably, administration of an IL-9-neutralizing antibody (anti-IL-9) inhibited CTL responses induced by CurDCs (Fig. 7g), indicating that Th9/IL-9 contributes to dectin-1-activated DC-induced CTL responses *in vivo*.

Finally to define the functional relationship between Th9/IL-9 and dectin-1-activated DC-induced antitumour effects, OT-II mice with established B16-OVA tumours were treated with OVA peptide-pulsed CurDCs in the presence or absence of anti-IL-9. We found that blockade of IL-9 with anti-IL-9 abolished the antitumour effects induced by dectin-1-activated DCs (Fig. 7h), demonstrating the important role of Th9/IL-9 in mediating the antitumour effects induced by dectin-1-activated DCs *in vivo*. Collectively, these data demonstrated that dectin-1-activated DCs mediated their antitumour effects via their ability to induce Th9 cells and IL-9 production *in vivo*.

Dectin-1-NF-κB signalling induced DC production of a specific profile of cytokines, chemokines and costimulatory surface molecules, including TNFSF15 and OX40L, but little IL-12, which favoured Th9 cell induction *in vitro* and *in vivo*. MyD88-mediated signals also activate NF-κB pathway. However, we found that lipopolysaccharide (LPS), a potent stimulator of MyD88/NF-κB signalling, only slightly upregulated OX40L expression in LPS-activated DCs (LpsDCs) as compared with CurDCs (Supplementary Fig. 13A). In addition, CurDCs but not LpsDCs could significantly upregulate IL-9 production and IRF4 expression in CD4^+^ T cells under Th9-polarizing condition (Supplementary Fig. 13B,C). Furthermore, CurDCs induced more potent inhibition on tumour growth than LpsDCs (Fig. 7h). One reason that might be contributed to this discrepancy is that MyD88-mediated signals potently stimulate DC expression of Th1-polarizing cytokine IL-12 (ref. 31). Taken together, these observations suggest that dectin-1-mediated signalling may be more efficient than MyD88-dependent signalling in promoting DC-induced antitumour Th9 cell differentiation *in vitro* and *in vivo*.

## Discussion

Th9 cells have been shown to mediate potent antitumour effects *in vivo*^7^,^8^; therefore, investigation of more efficient strategies to induce and expand Th9 cells *in vivo* may have high clinical significance in tumour immunotherapy. Dectin-1 signalling has been shown to induce Th17 and Th1 cell responses *in vivo*, which are essential for protecting a mammalian host from fungal infection^27^,^28^,^32^. However, whether dectin-1-activated DCs promote the development of antitumour Th9 cells remains unknown. In this study, we found that DCs activated by dectin-1 agonists potently promoted Th9 cell differentiation, and such Th9 cells expressed high levels of IL-9- and Th9-related transcription factor IRF4, but not Th1-, Th2- and Th17-related cytokines and transcription factors. This result was confirmed by using a dectin-1 blocking antibody or dectin-1^−/−^ DCs as they almost completely abrogated the capability of dectin-1 agonist-activated DCs in promoting Th9 cell induction. Importantly, in the functional tests, we found that dectin-1-activated DCs induced potently antitumour immunity against established tumours. Mechanistic studies revealed that immunization with dectin-1-activated DCs efficiently promoted the production of Th9 cells and IL-9, which were required for the antitumour effects induced by dectin-1-activated DCs. Thus, our data established the role for dectin-1 in activating DCs for the induction of potent Th9 cell responses against cancers *in vivo*.

Previous studies showed that dectin-1 activation in DCs induces the expression of TNF-α, IL-6, IL-23 and IL-12, which are related to Th17 and Th1 cell differentiation^24^,^27^,^32^,^33^,^34^. In this study, we identified other cytokines and costimulatory molecules, especially the TNF/receptor family members TNFSF15, OX40L, TNFSF8, TNFRSF26 and TNFRSF12a, that were upregulated by dectin-1 signalling and important for Th9 differentiation. Functional tests showed that dectin-1-induced overexpression of TNFSF15 and OX40L is responsible for the enhanced Th9 cell response induced by dectin-1-activated DCs. This result is consistent with previous observations that ligation of OX40 inhibits the production of regulatory T cells and Th17 cells and diverts CD4^+^ T cells to Th9 cells^11^. Interestingly, a recent study reported that TNFSF15 potently promotes Th9 differentiation and the pathogenicity of IL-9-dependent allergic diseases^35^. Thus, we identify TNF family members TNFSF15 and OX40L as key mediators for promoting Th9 cell differentiation primed by dectin-1-activated DCs. OX40L/OX40 were suggested to be unique in the TNF/receptor family in promoting Th9 cell induction^11^. In contrast, we and others found that TNFSF15 is also a powerful inducer of Th9 cells. Thus, other TNF family members may also be involved in the development of antitumour Th9 cells. Further studies will be necessary to investigate the function of other TNF/receptor family members expressed by dectin-1-activated DCs in the induction of Th9 cells and antitumour immunity.

Engagement of dectin-1 by β-glucan was shown to activate the transcription factor NF-κB through Syk- and Raf1-dependent signalling pathways, leading to the production of IL-6, IL-1β, IL-23 *et al*.^24^,^33^,^34^. However, the dectin-1 downstream signalling pathways responsible for TNFSF15 and OX40L expression were not defined. In this study, we found that dectin-1 agonists potently activated Syk, Raf1 and NF-κB signalling pathways in DCs, more powerful than regular DC maturation reagents TNF-α and IL-1β. Thus, we speculated that Syk, Raf1 and NF-κB signalling pathways triggered by dectin-1 might be responsible for TNFSF15 and OX40L expression. Indeed, we found that blocking Syk, Raf1 and NF-κB signalling by their specific inhibitors or siRNAs inhibited the expression of TNFSF15 and OX40L in dectin-1-activated DCs. Furthermore, we found that p52-RelB and p50-RelB dimmers directly bound to and activated *Tnfsf15* promoter, while p52-RelB, p50-RelB and p50 directly bound to and activated *Ox40l* promoter, providing direct evidence for NF-κB signalling in promoting TNFSF15 and OX40L expression. Thus, we identify Syk, Raf1 and NF-κB signalling pathways as key downstream pathways responsible for TNFSF15 and OX40L expression induced by dentin-1 in DCs. Nevertheless, we cannot exclude the possibility that some other factors that regulate TNFSF15 and OX40L expression might be induced through dectin-1-triggered Syk, Raf1 and NF-κB signalling pathways.

In this study, we found that dectin-1-activated DCs induced potent antitumour effects *in vivo*. However, cellular mechanisms underlying the antitumour effects were not determined. Dectin-1 agonists have been shown to induce Th17 and Th1 cell responses *in vivo*. In this study, we found that dectin-1-activated DCs induced potent Th9 cell responses *in vivo*. Th1 cells are a traditional antitumour Th subset^36^, while Th17 cells may also play a role in antitumour immunity^37^. Th9 cells were found to induce potent antitumour immunity in mouse models, better than Th1 and Th17 cells^7^,^8^. Based on these observations, we speculated that the antitumour effects of dectin-1-activated DCs *in vivo* mainly relied on Th9 cells and IL-9 induced by dectin-1-activated DCs. Indeed, we found that blockade of IL-9 with anti-IL-9 antibody abolished the antitumour effects induced by dectin-1-activated DCs. Thus, our data demonstrated that Th9 cells are the main effector cells in mediating the antitumour effects induced by dectin-1-activated DCs.

IL-9 expressed by dectin-1-activated DC-induced Th9 cells could have the potential to improve Th1/17/9 cell survival and proliferation *in vivo* and *in vitro*. In addition, curdlan may improve DC survival as compared with LPS, which exhibits detrimental effects on DC survival^38^. As our results showed that the improved efficacy of CurDCs to control the established tumour is dependent on IL-9, we believe that even if CurDC improved the T-cell activation, proliferation or survival or DC survival, Th9 induction by CurDCs played the most important role for the observed antitumour responses.

Neutralization of IL-9 was shown to promote melanoma tumour growth in C57BL/6 mice^7^,^8^. However, in this study, we observed no significant difference in B16-OVA tumour growth between mice received IL-9 neutralization antibodies and control IgG in either PBS-, BMDC- or LpsDC-treated groups. Reasons for this discrepancy could be that (i) we used OT-II mice in this study which largely lack CD8^+^ T cells, the important antitumour effector cells and (ii) the beginning of administration of anti-IL-9 antibodies was on day 4 after tumour challenge in this study versus day 0 in previous studies.

In summary, our study demonstrates dectin-1-activated DCs as a powerful inducer of antitumour Th9 cells *in vitro* and *in vivo* and identifies TNFSF15 and OX40L as key factors in mediating Th9 cell differentiation primed by dectin-1-activated DCs. Syk, Raf1 and NF-κB signalling pathways triggered by dectin-1 were required for dectin-1-induced OX40L and TNFSF15 expression. Finally, dectin-1-activated DCs induce potent therapeutic antitumour effects in mouse models, and the antitumour effects depended on induced Th9/IL-9. Our findings may have important clinical implications.

## Methods

### Mice and cell lines

C57BL/6, Balb/c, OX40^−/−^ (B6.129S4-Tnfrsf4tm1Nik/J) and OT-II (C57BL/6-Tg(TcraTcrb)425Cbn/J) mice were purchased from the Jackson Laboratory. Dectin-1^−/−^ mice were provided by G. Brown (University of Aberdeen, Aberdeen, Scotland), and the lack of dectin-1 expression in mouse DCs was confirmed by flow cytometry and qPCR (Supplementary Fig. 1). Mice were bred and maintained in pathogen-free facilities at the First Hospital Animal Center of Jilin University. Six-to-eight-week old mice were used for experiments. All animal experimental procedures were reviewed and approved by the Animal Ethical Committee of First Hospital of Jilin University.

B16 and B16-OVA melanoma cell lines (ATCC) were cultured in Iscove's modified Dulbecco's media (Invitrogen) supplemented with 10% heat-inactivated fetal bovine serum (FBS, Hyclone), 100 U ml^−1^ penicillin (Invitrogen) and 100 mg ml^−1^ streptomycin (Invitrogen). MPC-11 myeloma cells were cultured in RPMI 1640 supplemented with FBS (10%), L-glutamine (2 mM) and penicillin/streptomycin.

### Reagents

Recombinant mouse GM-CSF, TNF-α, IL-1β, IL-4, IL-9 and human TGF-β were purchased from Peprotech. TNFSF15 was purchased from R&D Systems. MHC class II restricted OT-II OVA peptide (OVA323–339, ISQAVHAAHAEINEAGR) was purchased from Genscript. Functional anti-mouse CD3e and CD28 antibodies (mAbs) were purchased from eBioscience. Dectin-1-blocking mAb was purchased from Invivogen. TNFSF15 neutralization mAb and OX40L-blocking mAb were purchased from R&D Systems. Curdlan and Scleroglucan were purchased from Sigma-Aldrich and Invivogen, respectively. Piceatannol (a Syk inhibitor) and GW5074 (a Raf1 kinase inhibitor) were purchased from Calbiochem. Bortezomib, a NF-κB inhibitor, was purchased from Selleckchem.

### Dendritic cell generation

BMDCs were generated as described previously^29^. In brief, BM cells (4 × 10^5^ ml^−1^) were cultured in RPMI 1640 complete medium supplemented with GM-CSF (20 ng ml^−1^) and IL-4 (10 ng ml^−1^). At day 4, culture medium was removed and replaced with fresh GM-CSF and IL-4-containing medium. At day 7, semi-adherent cells were collected as immature DCs (iDCs) and matured in fresh medium containing TNF-α (10 ng ml^−1^) and IL-1β (10 ng ml^−1^) (BMDCs). In some cultures, iDCs were matured with Curdlan (5 μg ml^−1^) or Scleroglucan (10 μg ml^−1^). Cells were matured for 48 h, and at day 9, semi-adherent cells were collected as mature DCs (mDCs) for further experiments or gene expression analysis by flow cytometry and qPCR.

In blocking experiments, iDCs were matured with TNF-α/IL-1β or Curdlan in the presence of a blocking anti-dectin-1 antibody (5 μg ml^−1^) or control IgG (5 μg ml^−1^) for 2 days. mDCs were collected for *in vitro* Th9 cell differentiation.

In some experiments, iDCs were generated from WT and dectin-1^−/−^ C57BL/6 mice and matured with TNF-α/IL-1β, Curdlan or Scleroglucan for 2 days. mDCs were collected for further experiments or gene expression analysis by flow cytometry and qPCR.

In some experiments, Piceatannol (40 μM), GW5074 (1 μM) or bortezomib (10 nM) was added to the medium during DC maturation, and cultures with addition of DMSO (0.1%; Sigma) served as control. Cells were matured for 48 h; and mDCs were collected and analysed by flow cytometry and qPCR. Cytokines in culture supernatants were assessed by ELISA.

### Flow cytometry

Flow cytometry analysis was performed as described previously^29^. Alex Fluor 700-, APC-, FITC-, PE-, PE-Cy7- or PerCP-conjugated mAbs against CD3 (cat #: 555274), CD4 (cat #: 552775), CD8 (cat #: 561093), CD25 (cat #: 553072), CD62L (cat #: 560517), CD11c (cat #: 558079), CD40 (cat #: 558695), CD80 (cat #: 553769), CD86 (cat #: 560581) and CD134 (OX40) (cat #: ab33998) were purchased from BD Biosciences. PE-labelled dectin-1 (CLEC7a) (cat #: 144304) and IL-9 (cat #: 514103) mAbs were purchased from Biolegend. PE-labelled CD252 (OX40L) mAb (cat #: 12-5905-82) was purchased from eBioscience. After staining, cells were analysed using a BD LSRFortessa flow cytometer.

Intracellular staining was performed by using a Cytofix/Cytoperm kit (BD Biosciences) according to the manufacturer's instruction. Cell samples were acquired and analysed by a BD LSRFortessa cytometer.

### *In vitro* Th9 cell differentiation

Naive CD4^+^ T cells (CD4^+^CD25^−^CD62L^hi^) were obtained from mouse spleen cells by fluorescence activated cell sorter (FACS) as previously described^8^,^10^. Naive CD4^+^ T cells (1 × 10^5^ per well) were cultured with BMDCs or dectin-1-activated DCs (1 × 10^5^ per well) in the presence of plate-bound anti-CD3 (2 μg ml^−1^) plus TGF-β (3 ng ml^−1^) and IL-4 (10 ng ml^−1^). Th0 cells were generated without addition of TGF-β and IL-4 in the culture medium. In some experiment a blocking anti-OX40L mAb (5 μg ml^−1^) or a neutralization anti-TNFSF15 mAb (5 μg ml^−1^) were added. After 3 days of culture, cells were collected and analysed for gene expression at the mRNA or protein levels by flow cytometry, qPCR and ELISA.

To examine the Th9-polarizing function of TNFSF15, CD4^+^ naive T cells (1 × 10^5^ per well) were cultured under Th0 or Th9 polarizing conditions in the presence of anti-CD3 and anti-CD28 (2 μg ml^−1^) with or without addition of TNFSF15 (50 ng ml^−1^) for 3 days. Cells and culture supernatants were collected and analysed for IL-9 expression by qPCR and ELISA.

To examine the function of OX40L–OX40 interaction in Th9 cell differentiation primed by dectin-1-activated DCs, CD4^+^ naive T cells (1 × 10^5^ per well) were isolated from WT or OX40^−/−^ C57BL/6 mice and cocultured with BMDC or CurDC (1 × 10^5^ per well) in the presence of plate-bound anti-CD3 with or without addition of Th9 polarizing cytokines TGF-β and IL-4 for 3 days. Cells and supernatants were analysed by qPCR and ELISA.

### *In vitro* differentiation of Th1/2/17/Treg cells

Sorted naive CD4^+^ T cells (1 × 10^5^ per well) were cultured with DCs (1 × 10^5^ per well) in the presence of plate-bound anti-CD3 (1 μg ml^−1^) plus soluble anti-CD28 (2 μg ml^−1^) and Th polarizing cytokines: IL-12 (4 ng ml^−1^) for Th1, IL-4 (10 ng ml^−1^) for Th2, IL-6 (20 ng ml^−1^) plus TGF-β (5 ng ml^−1^) for Th17 and TGF-β (5 ng ml^−1^) for Treg. IL-12 and IL-6 were purchased from eBioscience. After 3 days of culture, cells were collected and analysed for gene expression by qPCR.

### qPCR and western blot analyses

qPCR was performed as previously described^10^. Total RNA was extracted from cells by using an RNeasy Mini kit (Qiagen) according to the manufacturer's instructions. The mRNA levels of *Dectin1*, *Il9*, *Ifng*, *Il4*, *Il5*, *Il13*, *Il17*, *Tnfsf15*, *Ox40l*, *Spi1*, *Irf4*, *Tbx21, Gata3* and *Rorc* by Th cells or DCs were analysed. Expression was normalized to the expression of the house-keeping gene *Gapdh*. Primer sets used for these analyses are listed in Supplementary Table 1.

Western blot assay was performed as previously described^10^. Anti-mouse phosphorylated (p)-Syk, Syk, pRaf1, Raf1, p-IKKα/β, IκB-α, p65, p50, c-Rel, RelB, p52, β-actin and HDAC1 antibodies were purchased from Cell Signaling Technology (CST). RIPA Buffer (cat #: 9806) were purchased from CST. Nuclear extraction kit (cat #: 78833) was purchased from Thermo Scientific. Images have been cropped for presentation. Full-size images are presented in Supplementary Figs 14 and 15.

### Gene-expression profiling

Immature DCs were generated from Balb/c mice and matured with TNF-α/IL-1β or Curdlan for 48 h. mDCs were collected and stored in Trizol reagent (Invitrogen) at −80 °C. All samples were sent to OneArray (http://www.OneArray.com.cn/, Beijing, China) for transcription profiling via genome-wide microarrays, and the subsequent data analysis was also performed by OneArray.

### RNA interference

RNA interference was performed as previously described^10^. In brief, day 6 iDCs were transfected with 50 nM siRNA with transfection reagent DF4 (Dharmacon) according to the manufacturer's protocol. Silencing was confirmed at the mRNA levels by qPCR. On day 8, iDCs were matured with TNF-α/IL-1β or Curdlan for 48 h. On day 10, mDCs were collected and analysed by qPCR and flow cytometry staining. siRNAs used are listed in Supplementary Table 2.

### Luciferase reporter assays

The luciferase reporter vector pGL4.10, a control vector pGL4.74 and expression vectors for NF-κB molecules p50, p65, c-Rel, p52 and RelB were purchased from Addgene. A 2500-bp mouse *Ox40l* promoter and a 2500-bp mouse *Tnfsf15* promoter were separately inserted into pGL4.10 (mOx40l-pGL4.10 and mTnfsf15-pGL4.10). HEK293T cells were transiently transfected with mOx40l-pGL4.10 (0.25 μg per well), mTnfsf15-pGL4.10 (0.25 μg per well) or pGL4.74 (0.05 μg per well) and expression vectors (0.5 μg per well) for NF-κB molecules by Lipofectamine 2000 (Invitrogen). Promoter activity was measured with Dual-Luciferase Reporter Assay System (Promega) according to the manufacturer's instructions. Values are normalized to internal control and expressed as the mean±s.d. of relative luciferase units.

### *In vivo* functional tests for dectin-1-activated DCs

iDCs generated from WT or dectin-1^−/−^ C57BL/6 mice were matured with TNF-α (10 ng ml^−1^) plus IL-1β (10 ng ml^−1^) or Curdlan (5 μg ml^−1^) for 48 h, and pulsed with OT-II OVA peptide (OVA_323-339_; 10 μg ml^−1^) for additional 6 h. Treated DCs were collected for mouse immunization. In the therapeutic model, 1 × 10^5^ B16-OVA cells were injected subcutaneously into OT-II mice. On day 3 after tumour challenge, mice were randomly divided into groups and immunized subcutaneously with 1 × 10^6^ treated DCs. Mice were immunized twice (1 week apart). Mice injected with PBS served as controls. In some experiments, mice were given control IgG or neutralization anti-IL-9 antibody (αIL-9, 100 μg per mouse) every 3 days beginning 1 day after the first DC immunization. Tumour development was monitored over time. Tumour volume was calculated by the formula: 3.14 × (mean diameter)^3^/6. Mice were killed when the tumour diameter reached to the range between 1.5 and 2 cm.

OT-II mice were challenged with B16-OVA tumour cells and immunized with OVA-peptide-pulsed DCs as described above. On day 3 after DC immunizations, serum samples were collected and total leukocytes from tumour-derived LNs and spleens were restimulated with 5 μg ml^−1^ OVA323–339 peptide for 24 h. Cytokine expression was analysed by qPCR, ELISA and intracellular staining.

To test the capacity of dectin-1-activated DCs in priming therapeutic immunity against native tumour-specific antigens, a myeloma tumour model MPC-11 was used. MPC-11 cells secrete an Id protein (IgG2b) which is a native tumour-specific antigen. To enhance its immunogenicity, Id protein was conjugated to keyhole limpet hemocyanin (KLH, EMD Biosciences) as described previously^39^. iDCs generated from Balb/c mice were pulsed with Id-KLH (50 μg ml^−1^) for 6 h and matured with TNF-α/IL-1β or Curdlan for additional 48 h. MPC-11 cells (1 × 10^6^) were injected subcutaneously into Balb/c mice. On day 6 after tumour challenge, mice were grouped and immunized subcutaneously with 1 × 10^6^ Id-KLH-pulsed DCs. Mice were immunized twice (1 week apart) and tumour development were monitored over time. On day 3 after the second immunization, spleen cells were collected and re-stimulated with Id-KLH-pulsed BMDCs or CurDCs with addition of control IgG or αIL-9 for 48 h.

### Cytotoxicity assay

The cytotoxicity assay was performed as previously described^10^. In brief, Id protein secreted by MPC-11 cells was used as tumour antigen. Balb/c mice (*n*=3 per group) were immunized twice (1 week apart) with 1 × 10^6^ Id-KLH-pulsed BMDCs or CurDCs. Mice treated with PBS served as control. Mice were given control IgG or an IL-9 neutralizing antibody (αIL-9,100 μg per mouse) every 3 days beginning 1 day after the first DC immunization. On day 2 after the second immunization, spleen cells from the mice were re-stimulated with Id-KLH-pulsed BMDCs for 48 h and used as effector cells. MPC-11 cells labelled with 5 μM CFSE were used as target cells, whereas MOPC-315 labelled with 0.5 μM CFSE were used as non-target cells.

### Statistical analysis

The Student's *t*-test was used to compare various experimental groups. A *P* value of <0.05 was considered significant.

### Data availability

The microarray data of BMDCs and CurDCs are stored in the GEO repository and is accessible under the accession number GSE81111. All data are available within the article (as figure source data or Supplementary Information Files) and/or from the authors on request.

## Additional information

**How to cite this article:** Zhao, Y. *et al*. Dectin-1-activated dendritic cells trigger potent antitumour immunity through the induction of Th9 cells. *Nat. Commun.* 7:12368 doi: 10.1038/ncomms12368 (2016).

## Supplementary Material

Supplementary Information Supplementary Figures 1-15 and Supplementary Tables 1 and 2

## Figures and Tables

**Table 1:** The upregulated genes of cytokines, chemokines and costimulatory surface molecules in CurDCs versus BMDCs identified by gene expression profiling (GEP).

**Rank #**	**Gene symbol**	**Gene name**	**Probe intensity**		**Fold change**
			**CurDC**	**BMDC**	
1	*Edn1*	endothelin 1	80,627	532	151.4
2	*Ccl20*	chemokine (C–C motif) ligand 20	3,659	118	30.9
3	*Il33*	interleukin 33	55,127	2,098	26.3
4	*Tnfsf15*	tumor necrosis factor (ligand) superfamily, member 15	22,008	1,103	19.9
5	*Igf2*	insulin-like growth factor 2	60,620	3,056	19.8
6	*Ccl4*	chemokine (C–C motif) ligand 4	338,983	23,896	14.2
8	*Npy*	neuropeptide Y	11,170	848	13.2
9	*Tnf*	tumor necrosis factor	33,032	3,046	10.8
10	*Cd200*	CD200 antigen	11,305	1,457	7.8
11	*Hbegf*	heparin-binding EGF-like growth factor	48,247	6,431	7.5
13	*Ccl3*	chemokine (C–C motif) ligand 3	371,668	52,647	7.1
14	*Angptl2*	angiopoietin-like 2	6,362	997	6.4
16	*Inhba*	inhibin beta-A	134,914	21,926	6.2
17	*Penk*	preproenkephalin	8,547	1,590	5.4
18	*Il1a*	interleukin 1 alpha	209,483	46,270	4.5
21	*Csf1*	colony stimulating factor 1 (macrophage)	115,228	28,784	4.0
23	*Nrp1*	neuropilin 1	21,066	5,783	3.6
24	*Tnfsf8*	tumor necrosis factor (ligand) superfamily, member 8	5,670	1,561	3.6
25	*Tnfrsf26*	tumor necrosis factor receptor superfamily, member 26	18,171	5,168	3.5
27	*Cd70*	CD70 antigen	5,444	1,708	3.2
28	*Tnfsf4*	tumor necrosis factor (ligand) superfamily, member 4	55,588	17,733	3.1
29	*Ccl2*	chemokine (C–C motif) ligand 2	11,114	3,574	3.1
30	*Nrp1*	neuropilin 1	12,982	4,189	3.1
32	*Il1f9*	interleukin 1 family, member 9	8,432	2,888	2.9
33	*Il12b*	interleukin 12b	5,740	2,068	2.8
34	*Igfbp7*	insulin-like growth factor binding protein 7	4,935	1,799	2.7
35	*Mfge8*	milk fat globule-EGF factor 8 protein	219,136	81,811	2.7
37	*Tnfrsf12a*	tumor necrosis factor receptor superfamily, member 12a	7,312	2,880	2.5
38	*Pdgfa*	platelet-derived growth factor, alpha	12,496	4,976	2.5
40	*Il12a*	interleukin 12a	5,791	2,552	2.3
42	*Osm*	oncostatin M	43,502	21,367	2.0
