# Supplementary material for: Dectin-1-activated dendritic cells trigger potent antitumour immunity through the induction of Th9 cells
Source: Nat Commun. 2016 Aug 5;7:12368. doi: 10.1038/ncomms12368 (PMC4980454; doi:10.1038/ncomms12368)
Supplement: Supplementary Information — Supplementary Figures 1-15 and Supplementary Tables 1 and 2 [file ncomms12368-s1.pdf]

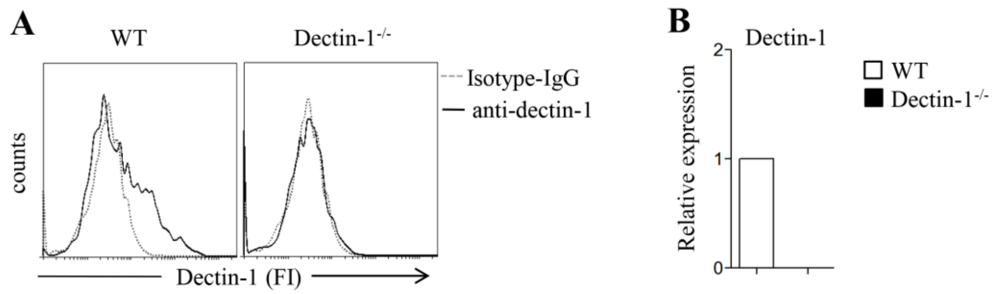


**Supplementary Figure 1**. Dectin-1 expression in iDCs. (A) Flow cytometry examined the expression of dectin-1 in mouse bone-marrow-derived iDCs generated from wildtype (WT, n=3) and dectin-1$^{-/-}$ mice (n=3). (B) qPCR assessed the mRNA levels of dectin-1 in WT and dectin-1$^{-/-}$ iDCs. Shown are representative results of 3 independent experiments.

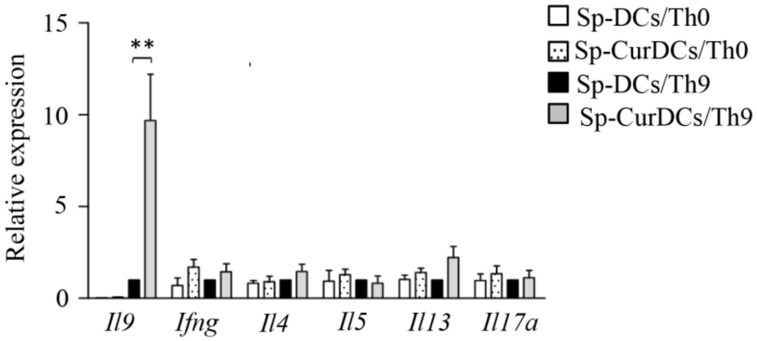


**Supplementary Figure 2.** Dectin-1-activated spleen DCs favor Th9 cell differentiation in vitro. Pooled (n=3) mouse spleen $CD11c^+$ cells isolated by fluorescence activated cell sorter were activated with (Sp-CurDCs) or without (Sp-DCs) Curdlan (5 μg/mL) in the presence of mouse GM-CSF (10 ng/mL) for 24 hours and cocultured with naïve $CD4^+$ T cells under Th0 or Th9 polarizing conditions for 3 days. qPCR assessed the mRNA levels of the indicated Th cytokines in $CD4^+$ T cells. Results shown are the mean ± SD of 3 independent experiments. **P < 0.05; **P < 0.01.*

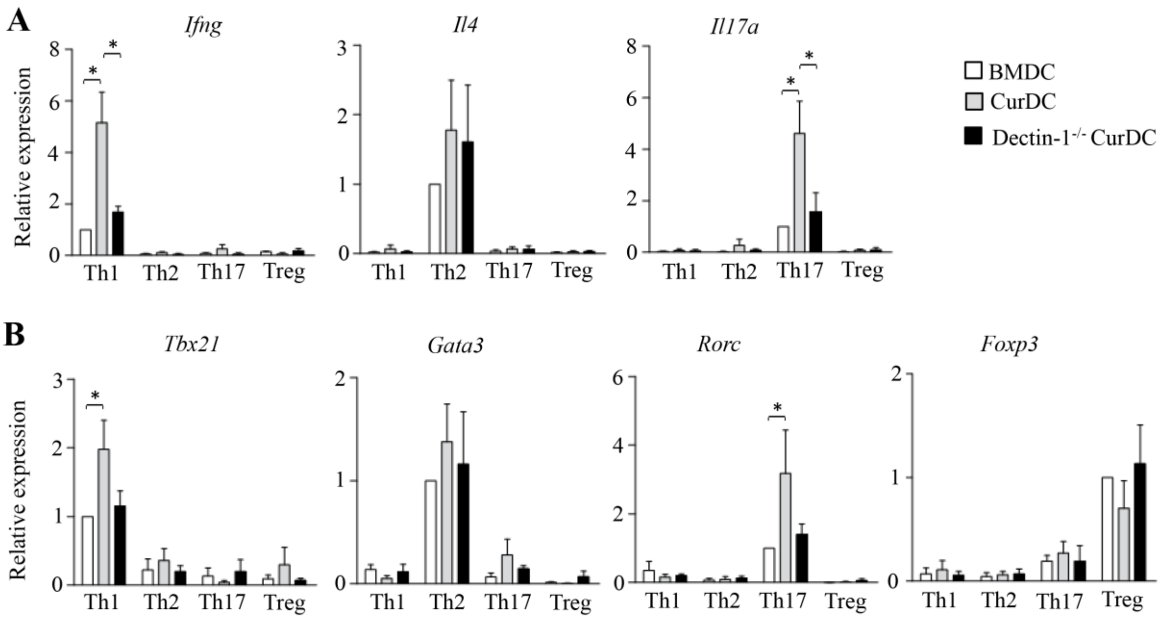


**Supplementary Figure 3.** Role of dectin-1 signaling in directing DCs for the differentiation of Th1/2/17/Treg cells in vitro. BMDC, CurDC or dectin-$1^{-/-}$ CurDC (n=3) were subjected to culture with naïve $CD4^+$ T cells under Th1, Th2, Th17 or Treg polarizing conditions. qPCR examined the expression of the indicated Th cytokines (A) and transcription factors (B) in $CD4^+$ T cells. Results shown are the mean ± SD of 3 independent experiments. **P < 0.05; **P < 0.01.*

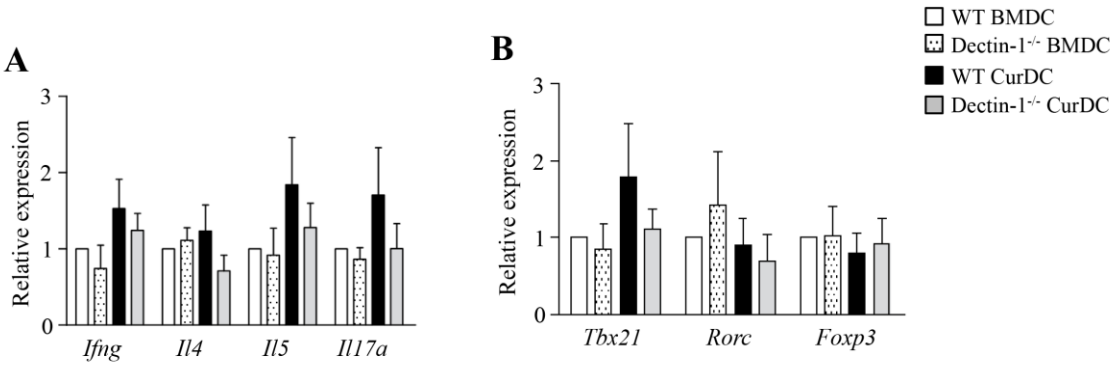


**Supplementary Figure 4.** Role of dectin-1 in CurDC-induced Th9 cell expression of Th1/2/17/Treg-related genes. BMDC and CurDC generated from WT or dectin-$1^{-/-}$ mice (n=2) were subjected to culture with naïve $CD4^{+}$ T cells under Th9 polarizing conditions. qPCR examined the expression of the indicated Th cytokines (A) and transcription factors (B) in $CD4^{+}$ T cells . Results shown are the mean ± SD of 3 independent experiments. *$^{*}P < 0.05$; $^{**}P < 0.01$.*

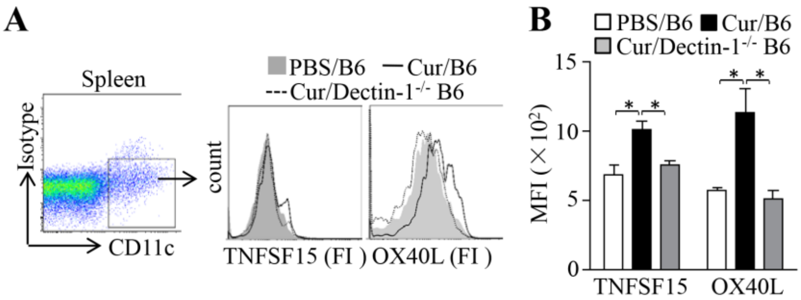


**Supplementary Figure 5.** Dectin-1 signaling stimulates DC expression of TNFSF15 and OX40L in vivo. B6 or Dectin-1$^{-/-}$ B6 mice (n=3 per group) were injected peritoneally with PBS or Curdlan (50 μg/mouse). (A) On day 2 after Curdlan injection, mouse spleen cells were collected and the expression of TNFSF15 and OX40L in CD11c$^{+}$ cells was analyzed by flow cytometry. (B) summary of results of three independent experiments obtained as in (A). MFI, mean fluorescence intensity. Results shown are the mean ± SD of 3 independent experiments. **P < 0.05; **P < 0.01.*

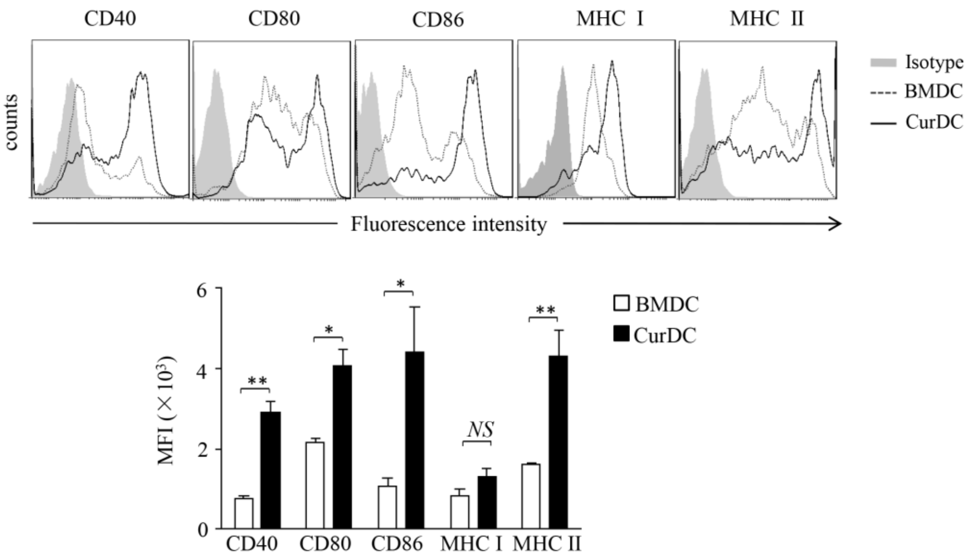


**Supplementary Figure 6.** Curdlan improves the phenotype of DCs. Mouse (n=3) iDCs were matured with TNF-α/IL-1β (BMDC) or Curdlan (CurDC). Cells were stained with the indicated antibodies and subjected to flow cytometry analysis. Below, summary of results of three independent experiments. **P < 0.05; **P < 0.01; NS, not significant.*

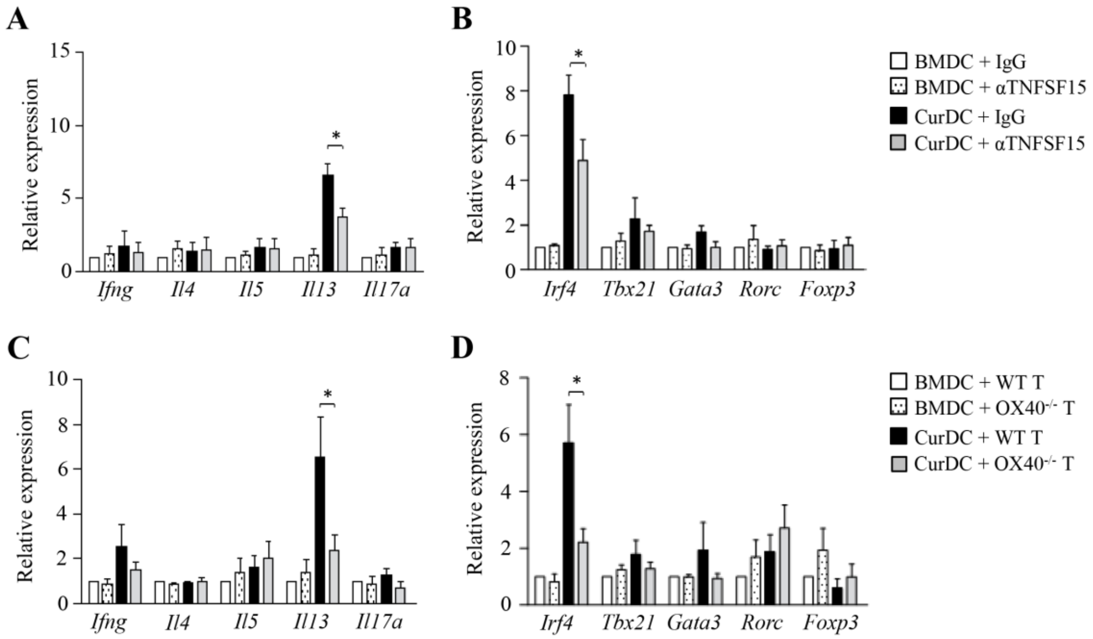


**Supplementary Figure 7.** Role of TNFSF15 and OX40L in CurDC-induced Th9 cell expression of Th9/1/2/17/Treg-related genes. Pooled mouse (n=3) splenic naïve $CD4^+$ T cells were cocultured with BMDCs or CurDCs under Th9 polarizing conditions with the addition of a TNFSF15-neutralization Ab (αTNFSF15) or control IgG. Cells were cultured for 3 days. qPCR examined the expression of the indicated Th cytokines (A) and transcription factors (B). Pooled mouse (n=3) splenic naïve $CD4^+$ T cells were isolated from WT or $OX40^{-/-}$ mice and differentiated under Th9 polarizing conditions in the presence of BMDCs or CurDCs for 3 days. qPCR examined the expression of the indicated Th cytokines (C) and transcription factors (D). Results shown are the mean ± SD of 3 independent experiments. *$*P < 0.05$; $**P < 0.01$.*

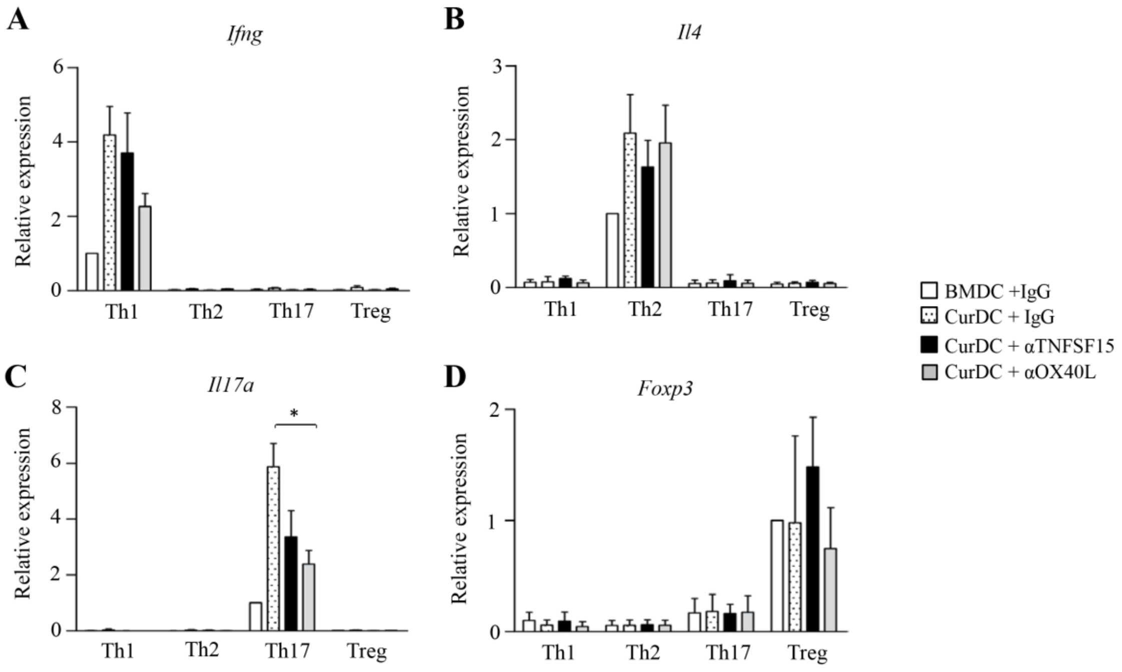


**Supplementary Figure 8.** Role of TNFSF15 and OX40L in mediating the differentiation of Th1/2/17/Treg cells primed by CurDCs. Pooled mouse (n=3) splenic naïve $CD4^+$ T cells were cultured with BMDCs or CurDCs under Th1, Th2, Th17 or Treg polarizing conditions with the addition of a TNFSF15-neutralization Ab (αTNFSF15) or control IgG. qPCR examined the expression of *Ifng* (A), *Il4* (B), *Il17a* (C) and *Foxp3* (D) in $CD4^+$ T cells . Results shown are the mean ± SD of 3 independent experiments. **P < 0.05; **P < 0.01*.

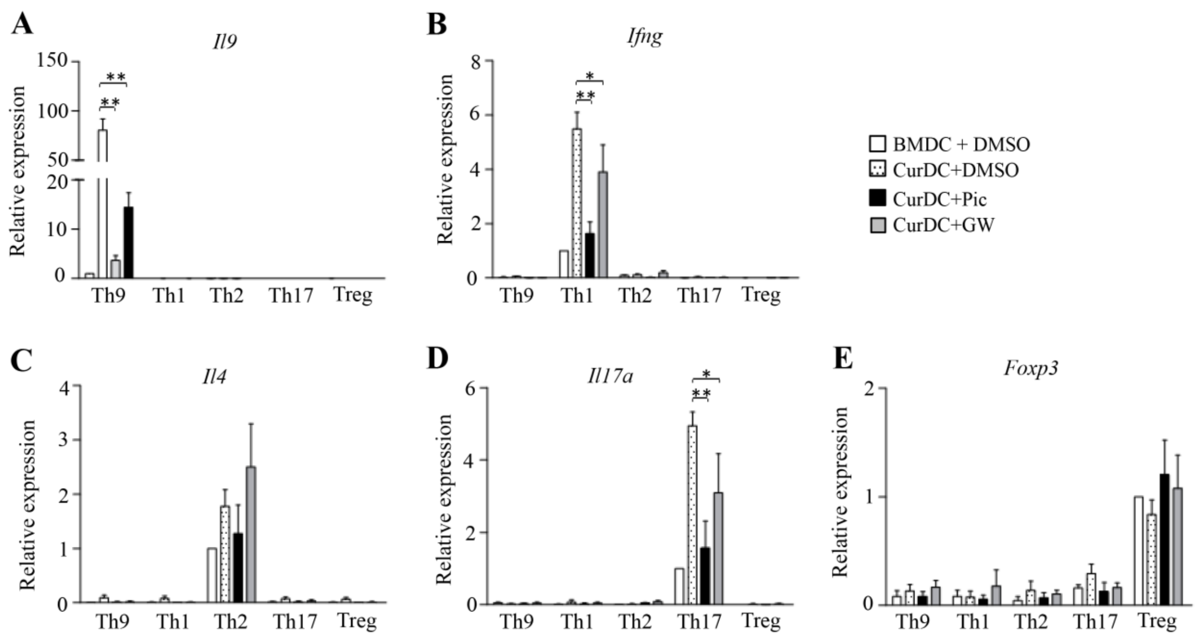


**Supplementary Figure 9.** Role of Syk/Raf-1 inhibitor-treated CurDCs in the differentiation of Th9/1/2/17/Treg cells. Mouse (n=3) iDCs were matured by TNFα/IL1β (BMDC) or Curdlan (CurDC) in the presence of piceatannol (Pic), GW5074 (GW) or DMSO as a control for 48 hours and cocultured with naïve $CD4^+$ T cells under Th9, Th1, Th2, Th17 or Treg polarizing conditions. qPCR examined the expression of *Il9* (A), *Ifng* (B), *Il4* (C), *Il17a* (D) and *Foxp3* (E) in $CD4^+$ T cells . Results shown are the mean ± SD of 3 independent experiments. $*P < 0.05; **P < 0.01$.

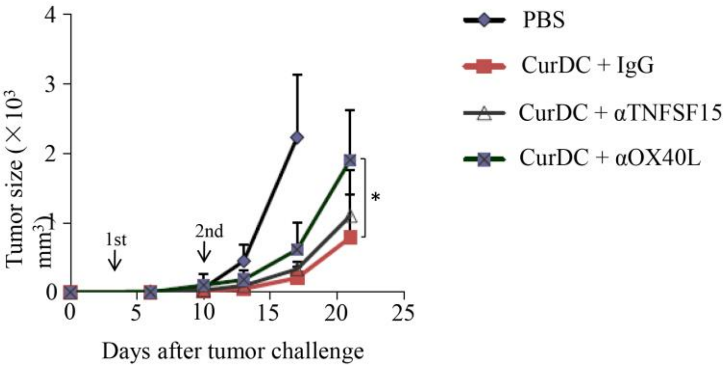


**Supplementary Figure 10.** Role of TNFSF15 and OX40L in dectin-1-activated DC-induced antitumor immunity. OT-II mice (n=5/group) were injected subcutaneously with $1 \times 10^5$ B16-OVA cells. On day 3 after tumor challenge, mice received two weekly immunizations with $1 \times 10^6$ OVA peptide-pulsed CurDCs plus TNFSF15 neutralization antibody (αTNFSF15, 100 μg/mouse), OX40L blocking antibody (αOX40L, 100 μg/mouse) or control IgG. Mice received PBS served as controls. Shown are the tumor growth curves. **P < 0.05; **P < 0.01.*

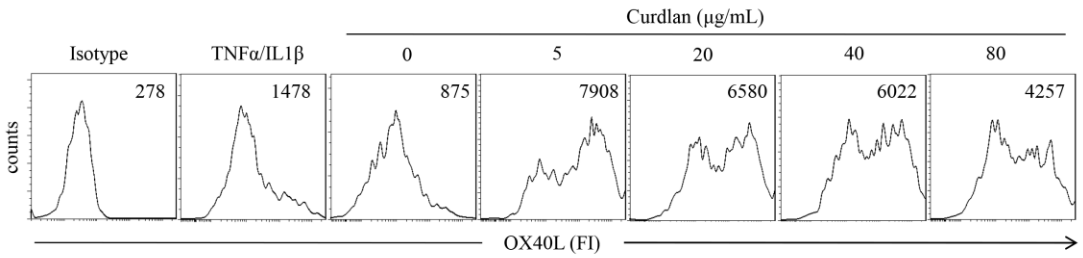


**Supplementary Figure 11.** Curdlan induced dose-dependent expression of OX40L in DCs. Mouse (n=3) iDCs were matured with TNF-α/IL-1β or Curdlan at the indicated dosages. Cells were stained with anti-OX40L antibody and subjected to flow cytometry analysis. Numbers in the histograms represent fluorescence intensity (FI) of the cells.

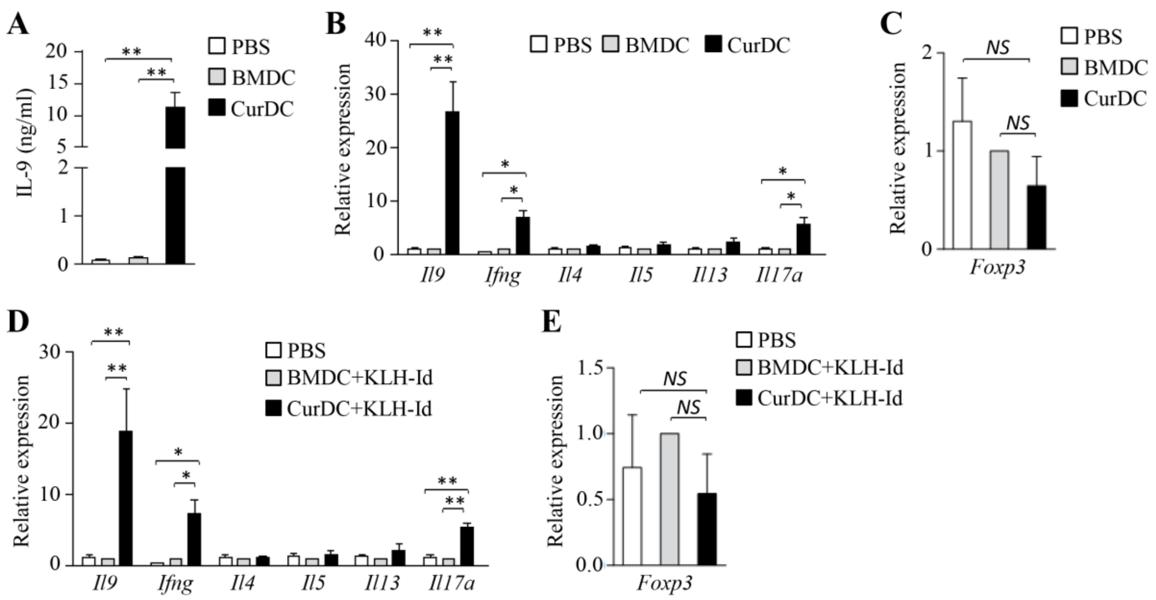


**Supplementary Figure 12.** Dectin-1-activated DCs induce Th9/IL-9 in vivo. OT-II mice (n=4-5/group) were immunized twice (one week apart) with OVA-peptide-pulsed BMDCs or CurDCs. PBS served as control. On day 3 after the 2$^{nd}$ immunization, total leukocytes from mouse spleens were harvested and CD4$^{+}$ T cells were separated by the Magnetic cell sorting (MACS). CD4$^{+}$ T cells were re-stimulated with OVA-peptide-pulsed DCs for 2 days. (A) ELISA examined IL-9 protein levels in the culture supernatants. (B) qPCR examined the expression of the indicated genes. (C) qPCR examined *Foxp3* expression. Balb/c mice were immunized twice (one week apart) with 1 $\times$ 10$^{6}$ KLH-Id-pulsed BMDCs or CurDCs. Mice received PBS served as controls. On day 3 after the 2$^{nd}$ immunization, CD4$^{+}$ T cells were re-stimulated with KLH-Id-pulsed BMDCs for 2 days. (D) qPCR examined the expression of the indicated genes. (E) qPCR examined *Foxp3* expression. Results shown are the mean ± SD of 3 independent experiments. **P < 0.05; **P < 0.01; NS, not significant.*

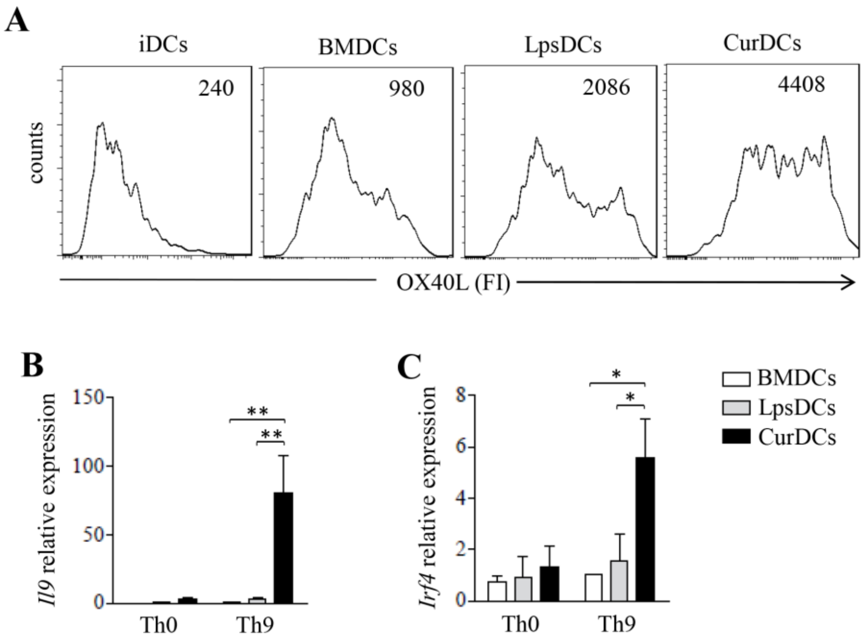


**Supplementary Figure 13.** Dectin-1-activated DCs are a more powerful inducer of Th9 cell differentiation than TLR4/MyD88-activated DCs. (A) Mouse (n=3) iDCs were matured with TNFα/IL1β (BMDCs), LPS (100 ng/mL, LPS-DCs) or Curdlan (10 μg/mL, CurDCs) for 48 hours. Unstimulated immature DCs (iDCs) were used as controls. Cells were stained with anti-OX40L antibody and subjected to flow cytometry analysis. Numbers in the histograms represent fluorescence intensity (FI) of the cells. (B&C) Mice (n=3) BMDCs, LPS-DCs or CurDCs were subjected to culture with naïve $CD4^+$ T cells under Th0 or Th9 (without addition of anti-CD28 Ab) polarizing conditions. qPCR examined the expression of *Il9* (B) and *Irf4* (C) in $CD4^+$ T cells. Results shown are the mean ± SD of 3 independent experiments. $*P < 0.05$; $**P < 0.01$.

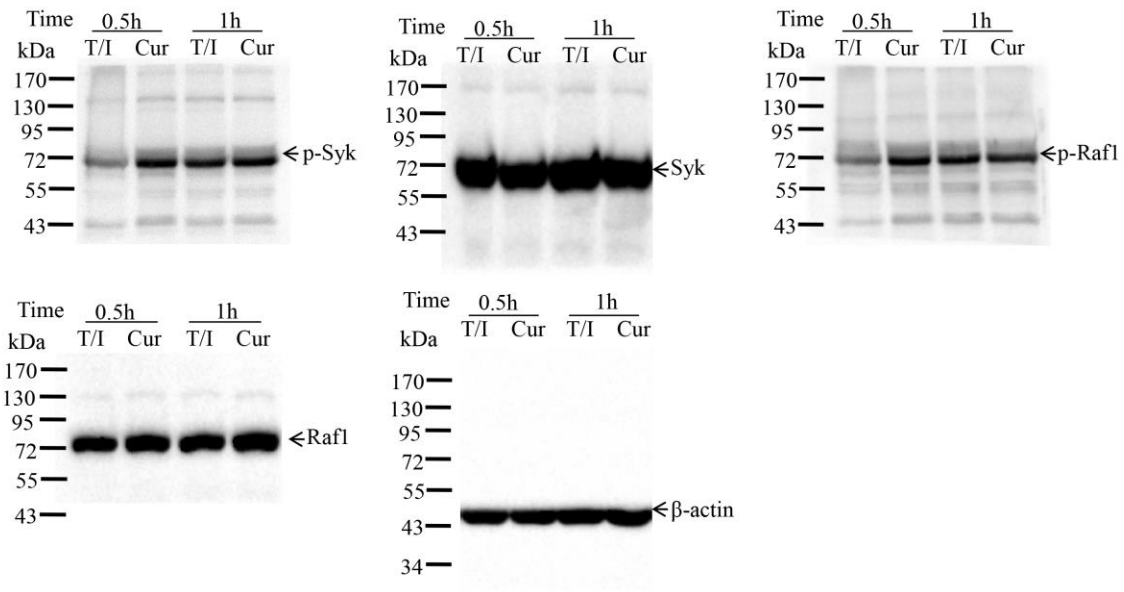


**Supplementary Figure 14A. Original gel images for Fig. 5A.**

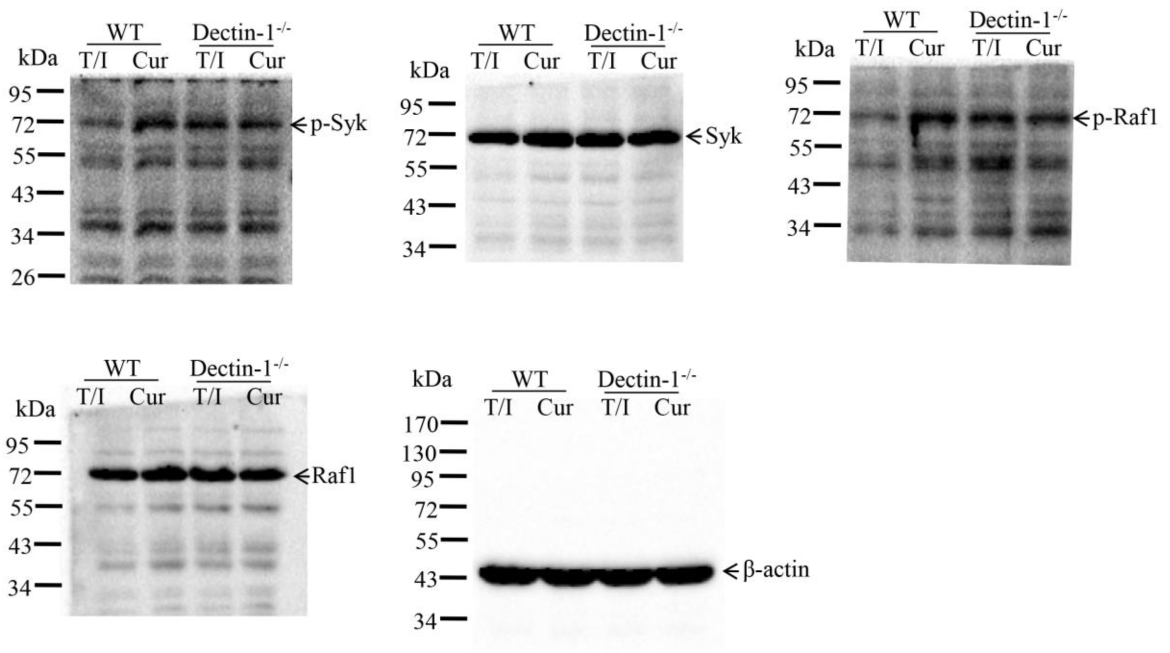


**Supplementary Figure 14B. Original gel images for Fig. 5B.**

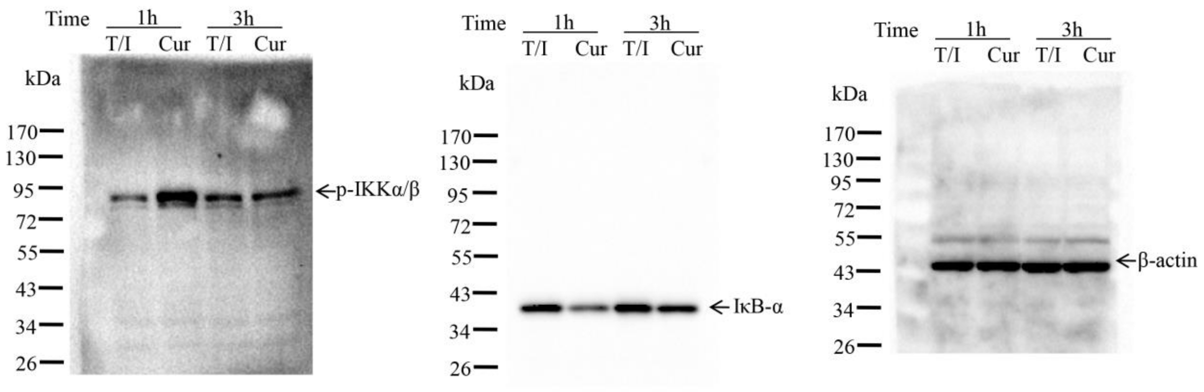


**Supplementary Figure 15A. Original gel images for Fig. 6A.**

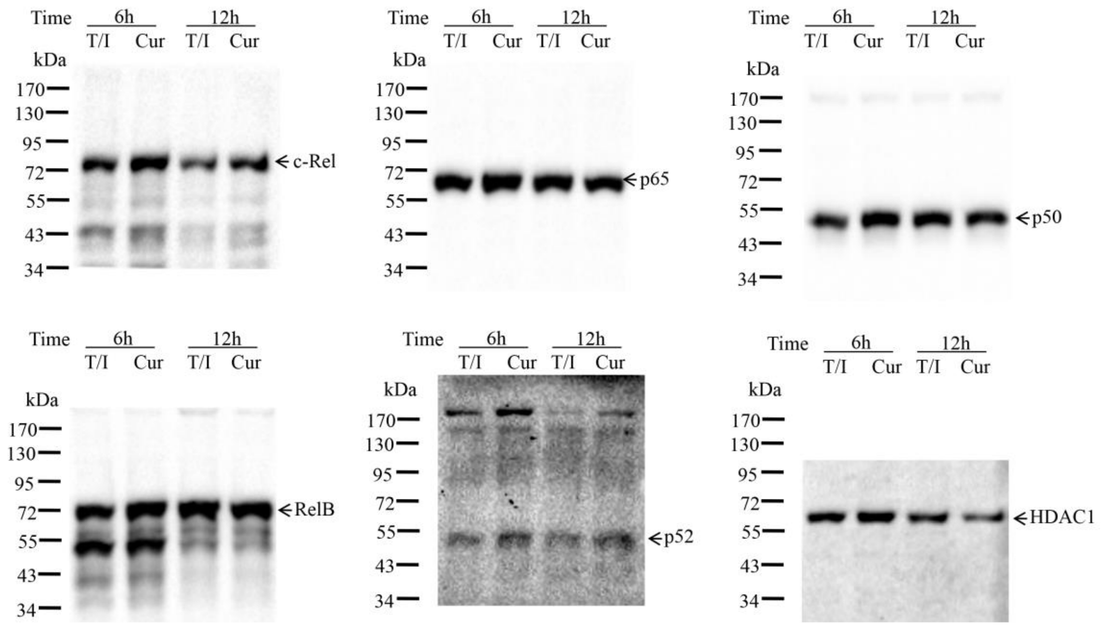


**Supplementary Figure 15B. Original gel images for Fig. 6B.**

**Supplementary Table 1.** Primer pairs used for quantitative real-time PCR (qPCR)

| Mouse genes | Sequences | |
|---|---|---|
| | **Forward** | **Reverse** |
| *dectin-1* | 5'-AGAACCACAAGCCCACAGAA | 5'-ATCCAATTAGGAAGGCAAGG |
| *Ifn-γ* | 5'-GCTTTGCAGCTCTTCCTCA | 5'-TTTCTTCCACATCTATGCCACT |
| *Il-4* | 5'-GAGCTCGTCTGTAGGGCTTC | 5'-TGCAGCTCCATGAGAACACT |
| *Il-5* | 5'-TGAGACGATGAGGCTTCCTG | 5'-AGCATTTCCACAGTACCCCC |
| *Il-9* | 5'-GTGTCTCTCCGTCCCAACTGATG | 5'-GATTTCTGTGTGGCATTGGTCAG |
| *Il-13* | 5'-GGCAGCATGGTATGGAGTGT | 5'-CTTGCGGTTACAGAGGCCAT |
| *Il-17* | 5'-TCCAGAAGGCCCTCAGACTA | 5'-TGAGCTTCCCAGATCACAGA |
| *Ox40l* | 5'-TCAGAGGAGCAGTTACCAGA | 5'-ACACTTGATGACAACCGAAT |
| *Tnfsf15* | 5'-GAAGGATGGCAGAGGAGC | 5'-CAACAGGCAGCAGGTGAG |
| *Gata-3* | 5'-GCCTGCGGACTCTACCATAA | 5'-AGGATGTCCCTGCTCTCCTT |
| *Irf4* | 5'-AGCCCAGCAGGTTCATAACT | 5'-CTGGCAACCATTTTCACAAG |
| *RORγ* | 5'-CTGCAAAGAAGACCCACACC | 5'-GGTGATAACCCCGTAGTGGA |
| *Sfpi1* | 5'-AACAGATGCACGTCCTCGAT | 5'-CAAGGTTTGATAAGGGAAGCA |
| *T-bet* | 5'-TCAACCAGCACCAGACAGAG | 5'-ATCCTGTAATGGCTTGTGGG |
| *GAPDH* | 5'-TGCACCACCAACTGCTTAGC | 5'-GGATGCAGGGATGATGTTCT |

**Supplementary Table 2.** siRNAs used for gene silencing in DCs

| Target genes | # | Sequences | |
|---|---|---|---|
| | | Sense | Anti-sense |
| *Raf-1* | 1 | 5'-CUGCUAAAUGGAUUUCGAU | 5'-UUGAGAUUAUUCCACCCGG |
| | 2 | 5'-GGAUCAAAUCCUACUCCUU | 5'-AUCGAAAUCCAUUUAGCAG |
| | 3 | 5'-GAUUGAGGAUGCAAUUCGA | 5'-UCGAAUUGCAUCCUCAAUC |
| *Syk* | 1 | 5'-CCAUCGAGAGGGAACUUAA | 5'-UUAAGUUCCCUCUCGAUGG |
| | 2 | 5'-CCGGGUGGAAUAAUCUCAA | 5'-UUGAGAUUAUUCCACCCGG |
| | 3 | 5'-GGAUCAAAUCCUACUCCUU | 5'-AAGGAGUAGGAUUUGAUCC |
| | 4 | 5'-GCAGCAGAACAGGCACAUU | 5'-AAUGUGCCUGUUCUGCUGC |
| *Control* | 1 | 5'-UUCUCCGAACGUGUCACGU | 5'-ACGUGACACGUUCGGAGAA |
